# Supplementary material for: Wnt4 and ephrinB2 instruct apical constriction via Dishevelled and non-canonical signaling
Source: Nat Commun. 2023 Jan 20;14:337. doi: 10.1038/s41467-023-35991-6 (PMC9860048; doi:10.1038/s41467-023-35991-6)
Supplement: Supplementary file 1 — Supplementary Information [file 41467_2023_35991_MOESM1_ESM.pdf]

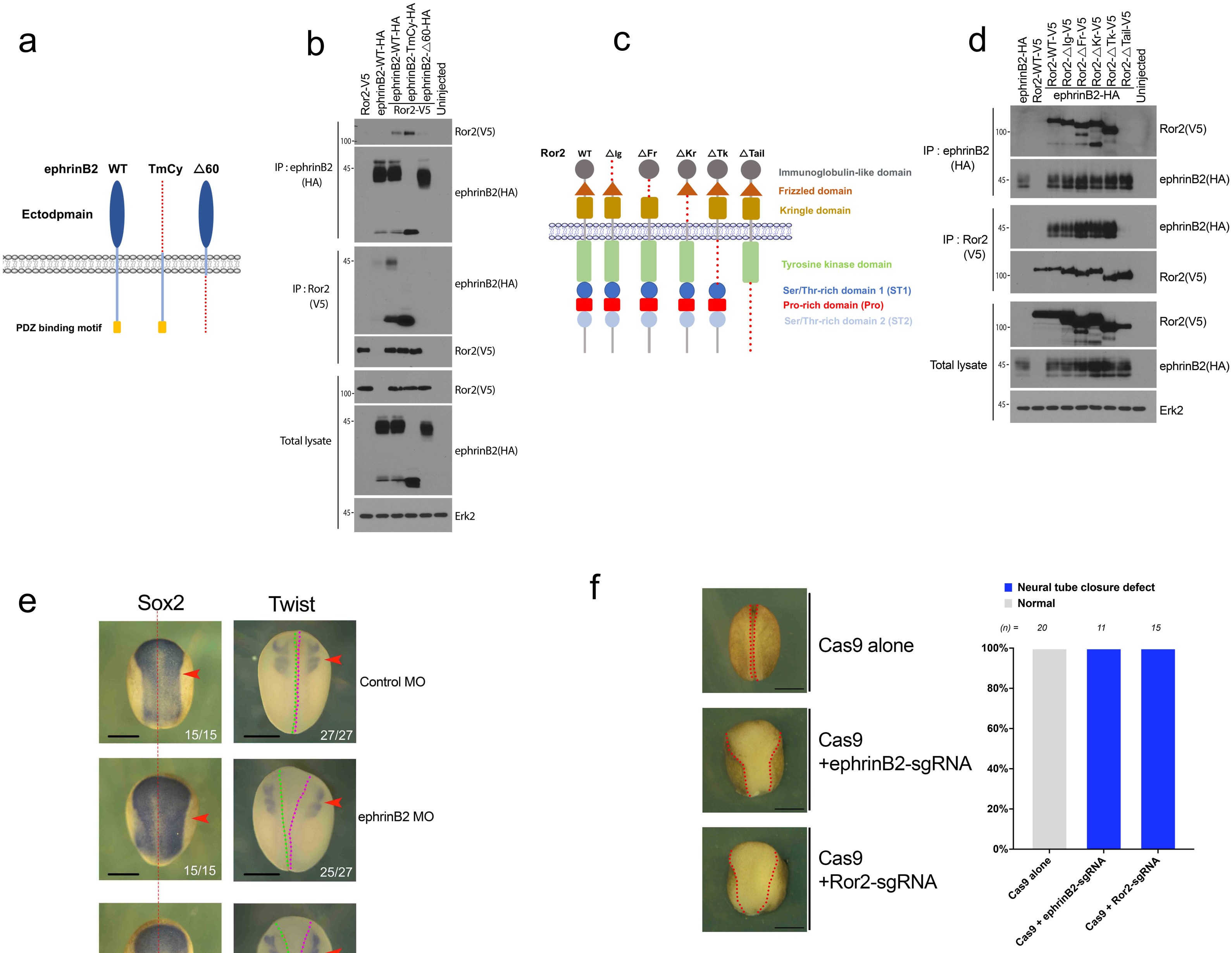

Target gene	location	genomic DNA sequences
ephrinB2	Exon 1	CGAAACGTTGCGTTTGGTCAGTGATAGTTTCTTGGGGCGGCCAGATCATGGCACTGA GCGGGGACTCGGTGTGGAAGTACAGCCTGGGAGTTTGTATTCTCTTGCTCAGGACTG CGATCTCCTGGTCTACAGTGCTGGATCCTATATACTGGAACTCGTCCAACGCCAGGT AAGTCCCGAGCCCTTTTACTCTTTACGTCAGTGTGTGAGGGCCACATACAGACACGG TGCGACACTCTAATCAGCAGTGTTGTATAGATATTATCAGCGTGTAAGCGTGTCCGT
Ror2	Exon 3	GGCCTGTCTTCTCCAACCGTGATCTGACCCTTTTTGAATTCTTCTTTCTCCCCAGGAT ACTATTTGACGTTTCTAGAACCTGTGAACAATATCACAATTGTGCAAGGTCAGGCAG CAACACTCCATTGCAAAGTGTCCGGGAATCCACTCCCCAATGTAAAGTGGCTGAAG AACGATGCACCTGTGGTTCAGGAGCCCAAGAGAATTACCATTAGGAAAACAGACTA CGGCTCACGATTACGGATACAAGACCTGGACACCACCGACACTGGCTATTATCAGT GTGTCGCCACCAATGGGATAAAGACCATTACTGCAACTGGAGTCCTCTTTGTTAGGC TTGGTAAGCTTTACTGTGCATTATAATTAAATAACAGACATGGCAAAGGAAGTAGTT

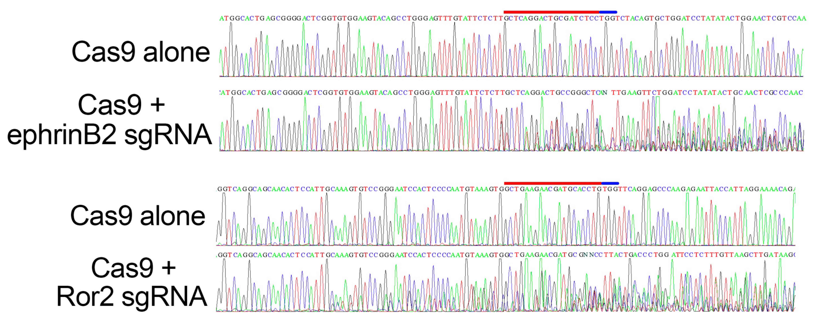

Supplementary Fig. 1. Ror2 interacts with ephrinB2 and regulates neural tube closure.

(a) Schematic of ephrinB2 deletion mutants. (b) Co-IP with Ror2 and the ephrinB2 deletion mutants at stage 13 embryos. (c) Schematic of Ror2 conserved domain deletion mutants. (d) Co-IP with ephrinB2 and the Ror2 conserved domain deletion mutants at stage 13 embryos. (e) *in situ* hybridization against the neuronal cell marker, *Sox2* and cranial neural crest marker, *Twist*. MOs were injected into D1.1 blastomere at the 16-cell stage embryos. Red dot lines indicate the midline of neural plate. The lateral boundaries of the neural plate are marked by dot lines (green; uninjected side and magenta; injected side). Red arrow heads indicate injected side. Number of embryos showing representative data/total number of analyzed embryos. Scale bar, 500μm. (f) Representative embryo phenotypes of ephrinB2 or Ror2 knockout embryos. See also Supplementary Movie. 2. Histogram depicts quantification of the phenotypes. The lateral boundaries of the neural plate are marked by red dot lines. Scale bar, 500μm. Genomic DNA information for CRISPR/Cas9 knockout. Red indicates the sgRNA target site, Intron in gray, exon in black, and PAM sequences in blue. Confirmation of ephrinB2 or Ror2 genetic disruption by DNA-sequencing of the amplified genomic DNA from F0 knockout embryos.

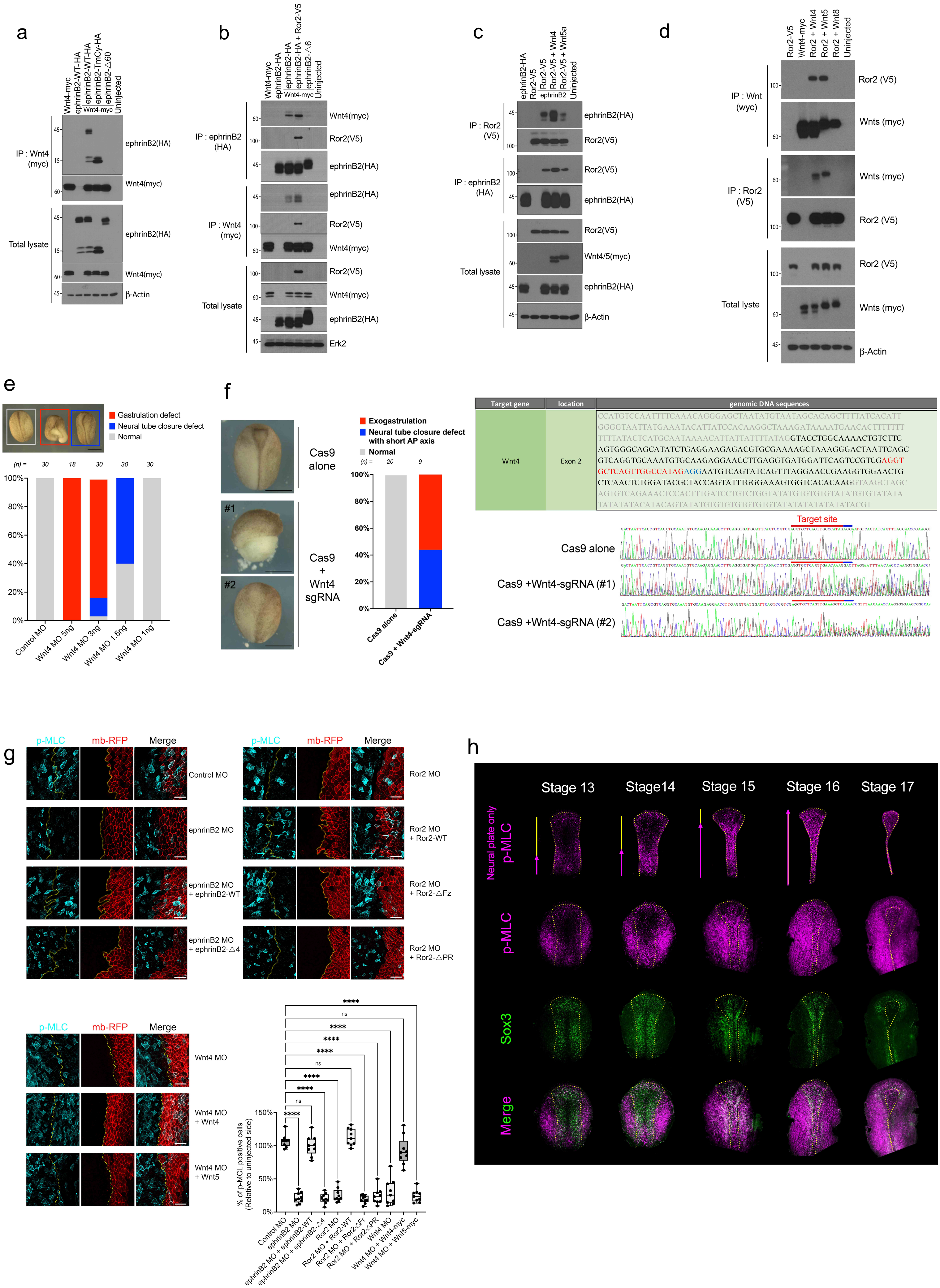
a
Wnt4-myc
ephrinB2-WT-HA
ephrinB2-WT-HA
ephrinB2-TmCy-HA
ephrinB2-△60
Uninjected
Wnt4-myc
IP : Wnt4 (myc)
Total lysate
ephrinB2(HA)
Wnt4(myc)
β-Actin
b
Wnt4-myc
ephrinB2-HA
ephrinB2-HA
ephrinB2-HA + Ror2-V5
ephrinB2-△6
Uninjected
IP : ephrinB2 (HA)
IP : Wnt4 (myc)
Total lysate
Wnt4(myc)
Ror2(V5)
ephrinB2(HA)
Erk2
c
ephrinB2-HA
Ror2-V5
Ror2-V5
Ror2-V5 + Wnt4
Ror2-V5 + Wnt5a
Uninjected
ephrinB2
IP : Ror2 (V5)
IP : ephrinB2 (HA)
Total lysate
Wnt4/5(myc)
β-Actin
d
Ror2-V5
Wnt4-myc
Ror2 + Wnt4
Ror2 + Wnt5
Ror2 + Wnt8
Uninjected
IP : Wnt (wyc)
IP : Ror2 (V5)
Total lyste
Ror2 (V5)
Wnts (myc)
e
Gastrulation defect
Neural tube closure defect
Normal
(n) = 30 18 30 30 30
Control MO
Wnt4 MO 5ng
Wnt4 MO 3ng
Wnt4 MO 1.5ng
Wnt4 MO 1ng
f
Cas9 alone
Cas9 + Wnt4 sgRNA
#1
#2
Exogastrulation
Neural tube closure defect with short AP axis
Normal
(n) = 20 9
Cas9 alone
Cas9 + Wnt4-sgRNA
Target gene
location
genomic DNA sequences
Wnt4
Exon 2
Target site
Cas9 alone
Cas9 +Wnt4-sgRNA (#1)
Cas9 +Wnt4-sgRNA (#2)
g
p-MLC
mb-RFP
Merge
Control MO
ephrinB2 MO
ephrinB2 MO + ephrinB2-WT
ephrinB2 MO + ephrinB2-△4
Ror2 MO
Ror2 MO + Ror2-WT
Ror2 MO + Ror2-△Fz
Ror2 MO + Ror2-△PR
Wnt4 MO
Wnt4 MO + Wnt4
Wnt4 MO + Wnt5
% of p-MCL positive cells (Relative to uninjected side)
ns
****
Control MO
ephrinB2 MO
ephrinB2 MO + ephrinB2-WT
ephrinB2 MO + ephrinB2-△4
Ror2 MO
Ror2 MO + Ror2-WT
Ror2 MO + Ror2-△Fr
Ror2 MO + Ror2-△PR
Wnt4 MO
Wnt4 MO + Wnt4-myc
Wnt4 MO + Wnt5-myc
h
Stage 13
Stage14
Stage 15
Stage 16
Stage 17
Neural plate only p-MLC
p-MLC
Sox3
Merge

Supplementary Fig. 2. Wnt4 interacts with ephrinB2 and regulates apical constriction.

(a) Co-IP with Wnt4 and the ephrinB2 deletion mutants (See Supplementary Fig. 1a) at stage 13 embryos. (b) Co-IP with Wnt4 and the ephrinB2 in the presence of exogenously expressed Ror2. (c) Co-IP with Ror2 and the ephrinB2 in the presence of exogenously expressed Wnt4 or Wnt5. (d) Co-IP with Ror2 and Wnt ligands, Wnt4, 5, and 8. (e) Representative embryo morphologies by Wnt4 knockdown. The histogram depicts the quantification of the phenotypes. MOs were injected into D1.1 blastomere at the 16 cell stage embryos. Scale bar, 500μm (f) Representative embryo phenotypes of Wnt4 knockout embryos. See also Supplementary Movie. 5. Histogram depicts quantification of the phenotypes. Genomic DNA information for CRISPR/Cas9 knockout. Red indicates the sgRNA target site, Intron in gray, exon in black, and PAM sequences in blue. Confirmation of Wnt4 genetic disruption by DNA-sequencing of the amplified genomic DNA from F0 knockout embryos in #1 and #2. Scale bar, 500μm. (g) phospho-MLC levels in neural plates. The box plot shows Min to Max with all data points. Numbers of neural plates/experiments: 9/3 for each column. One-way ANOVAs followed by Dunnett's multiple comparison tests were used. n.s., not significant, ****$p < 0.0001$. Scale bar, 50μm. (h) Immunostaining of phospho-MLC in developing embryos from stage 13 to 17. Sox3 indicates neural plate cells. The yellow dotted lines indicate the border of the neural plate. Scale bar, 500μm.

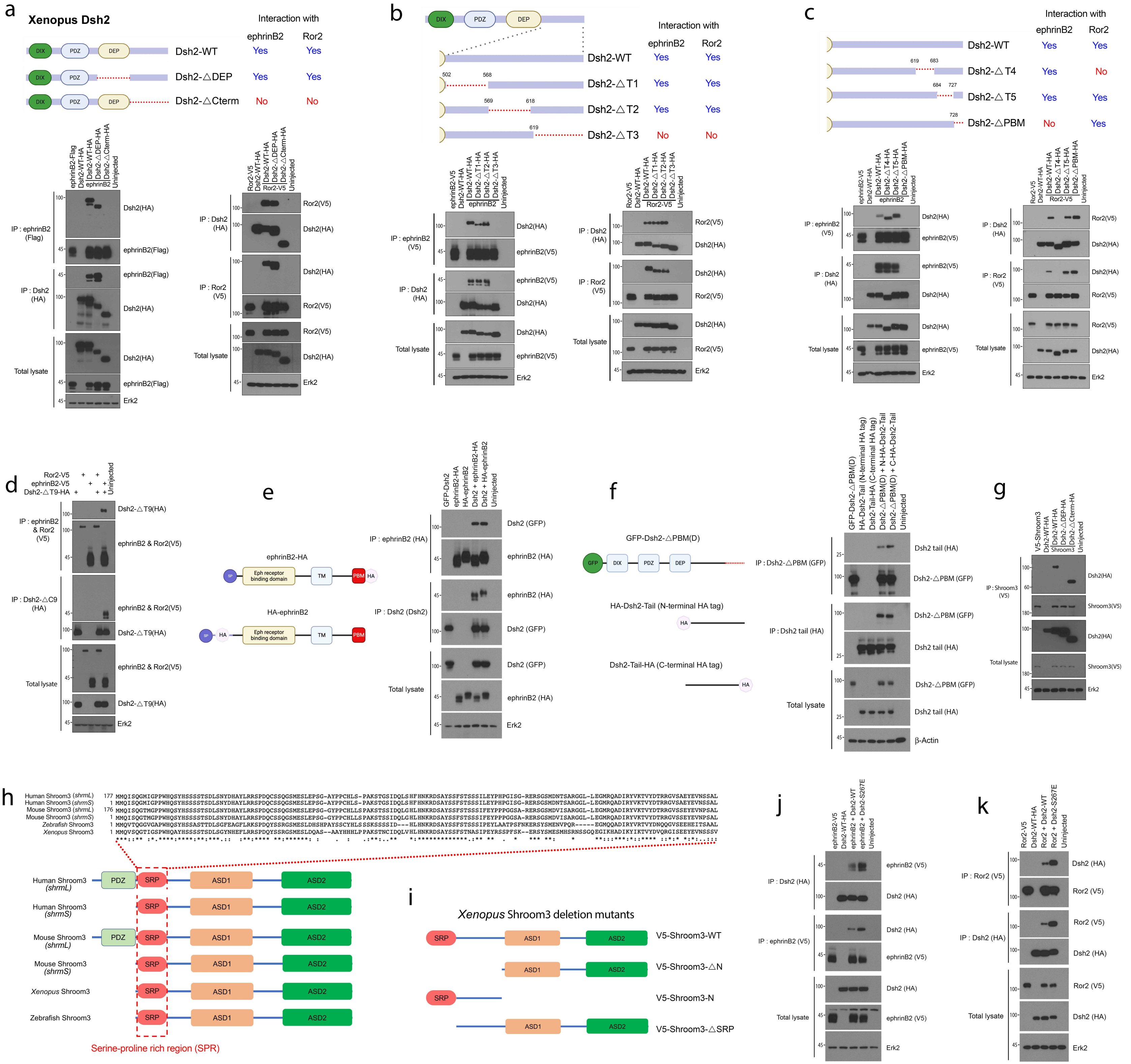
a
Xenopus Dsh2
Interaction with
ephrinB2
Ror2
Dsh2-WT
Yes
Yes
Dsh2-△DEP
Yes
Yes
Dsh2-△Cterm
No
No
DIX
PDZ
DEP
ephrinB2-Flag
Dsh2-WT-HA
Dsh2-△DEP-HA
Dsh2-△Cterm-HA
Uninjected
ephrinB2
IP : ephrinB2 (Flag)
IP : Dsh2 (HA)
Total lysate
Dsh2(HA)
ephrinB2(Flag)
Erk2
Ror2-V5
Ror2-V5
IP : Dsh2 (HA)
IP : Ror2 (V5)
Ror2(V5)
b
Dsh2-WT
Dsh2-△T1
Dsh2-△T2
Dsh2-△T3
502
568
569
618
619
ephrinB2-V5
Dsh2-△T1-HA
Dsh2-△T2-HA
Dsh2-△T3-HA
IP : ephrinB2 (V5)
ephrinB2(V5)
c
Dsh2-△T4
Dsh2-△T5
Dsh2-△PBM
683
684
727
728
Dsh2-△T4-HA
Dsh2-△T5-HA
Dsh2-△PBM-HA
d
Ror2-V5
ephrinB2-V5
Dsh2-△T9-HA
IP : ephrinB2 & Ror2 (V5)
IP : Dsh2-△C9 (HA)
Dsh2-△T9(HA)
ephrinB2 & Ror2(V5)
e
ephrinB2-HA
HA-ephrinB2
SP
Eph receptor binding domain
TM
PBM
HA
GFP-Dsh2
Dsh2 + ephrinB2-HA
Dsh2 + HA-ephrinB2
IP : ephrinB2 (HA)
IP : Dsh2 (Dsh2)
Dsh2 (GFP)
ephrinB2 (HA)
f
GFP-Dsh2-△PBM(D)
HA-Dsh2-Tail (N-terminal HA tag)
Dsh2-Tail-HA (C-terminal HA tag)
GFP
HA-Dsh2-Tail (N-terminal HA tag)
Dsh2-Tail-HA (C-terminal HA tag)
Dsh2-△PBM(D) + N-HA-Dsh2-Tail
Dsh2-△PBM(D) + C-HA-Dsh2-Tail
IP : Dsh2-△PBM (GFP)
IP : Dsh2 tail (HA)
Dsh2 tail (HA)
Dsh2-△PBM (GFP)
β-Actin
g
V5-Shroom3
Shroom3
IP : Shroom3 (V5)
Shroom3(V5)
h
Human Shroom3 (shrmL)
Human Shroom3 (shrmS)
Mouse Shroom3 (shrmL)
Mouse Shroom3 (shrmS)
Zebrafish Shroom3
Xenopus Shroom3
SRP
ASD1
ASD2
Serine-proline rich region (SPR)
i
Xenopus Shroom3 deletion mutants
V5-Shroom3-WT
V5-Shroom3-△N
V5-Shroom3-N
V5-Shroom3-△SRP
j
ephrinB2 + Dsh2-WT
ephrinB2 + Dsh2-S267E
IP : Dsh2 (HA)
IP : ephrinB2 (V5)
k
Ror2 + Dsh2-WT
Ror2 + Dsh2-S267E
IP : Ror2 (V5)
IP : Dsh2 (HA)
Dsh2 (HA)
Ror2 (V5)

Supplementary Fig. 3. Dsh2 mediates the formation of the WERDS protein complex.

(a) Schematic of Dsh2 conserved domain deletion mutants. Left: Co-IP assay with ephrinB2 and the Dsh2 deletion mutants at Stage 13 *Xenopus* embryos. Right: Co-IP assay with Ror2 and the Dsh2 deletion mutants. (b) Schematic of Dsh2 C-terminal region deletion mutants. Left: Co-IP assay with ephrinB2 and the Dsh2 deletion mutants. Right: Co-IP assay with Ror2 and the Dsh2 deletion mutants. (c) Schematic of Dsh2 C-terminal region deletion mutants. Left: Co-IP assay with ephrinB2 and the Dsh2 deletion mutants. Right: Co-IP assay with Ror2 and the Dsh2 deletion mutants. (d) Co-IP assay with Dsh2-ΔT9 and ephrinB2, Ror2. (e) Co-IP assay with GFP-Dsh2 and N- or C-terminal HA tagged ephrinB2. (f) Co-IP assay with GFP-Dsh2-ΔPBM and N- or C-terminal HA tagged Dsh2-tail. (g) Co-IP assay with Shroom3 and Dsh2 conserved domain deletion mutants. See also Supplementary Fig. 3a. (h) a multiple sequence alignment of shroom3 across different species (Human, Mouse, *Zebrafish*, and *Xenopus*). PDZ; PDZ domain, ASD1 or 2; Apx/Shroom domains 1 or 2. The red dotted line box indicates the serine-proline rich region (SPR). (i) Schematic of *Xenopus* Shroom3 deletion mutants. (j) Co-IP assay with ephrinB2 and WT-Dsh2-HA or Dsh2-S267E-HA mutant. (k) Co-IP assay with Ror2 and WT-Dsh2-HA or Dsh2-S267E-HA mutant.

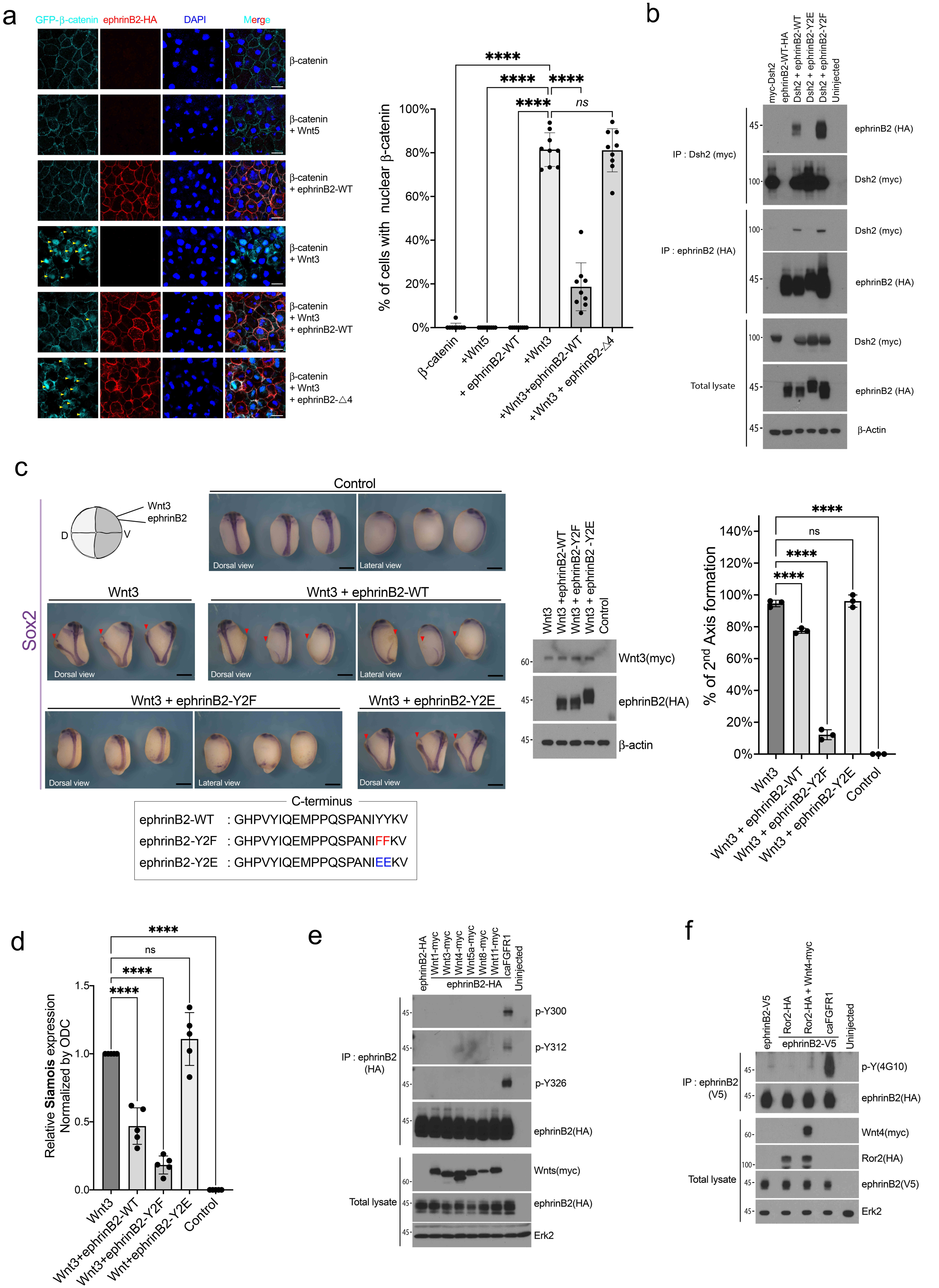
a
GFP-β-catenin
ephrinB2-HA
DAPI
Merge
β-catenin
β-catenin + Wnt5
β-catenin + ephrinB2-WT
β-catenin + Wnt3
β-catenin + Wnt3 + ephrinB2-WT
β-catenin + Wnt3 + ephrinB2-△4
% of cells with nuclear β-catenin
100%
80%
60%
40%
20%
0%
****
****
****
****
ns
β-catenin
+Wnt5
+ ephrinB2-WT
+Wnt3
+Wnt3+ephrinB2-WT
+Wnt3 + ephrinB2-△4
b
myc-Dsh2
ephrinB2-WT-HA
Dsh2 + ephrinB2-WT
Dsh2 + ephrinB2-Y2E
Dsh2 + ephrinB2-Y2F
Uninjected
IP : Dsh2 (myc)
IP : ephrinB2 (HA)
Total lysate
ephrinB2 (HA)
Dsh2 (myc)
β-Actin
45
100
c
Control
Wnt3
ephrinB2
D
V
Sox2
Wnt3 + ephrinB2-WT
Wnt3 + ephrinB2-Y2F
Wnt3 + ephrinB2-Y2E
Dorsal view
Lateral view
C-terminus
ephrinB2-WT : GHPVYIQEMPPQSPANIYYKV
ephrinB2-Y2F : GHPVYIQEMPPQSPANIFFKV
ephrinB2-Y2E : GHPVYIQEMPPQSPANIEEKV
Wnt3
Wnt3 +ephrinB2-WT
Wnt3 + ephrinB2-Y2F
Wnt3 + ephrinB2 -Y2E
Control
Wnt3(myc)
ephrinB2(HA)
β-actin
60
45
% of 2nd Axis formation
140%
120%
100%
80%
60%
40%
20%
0%
****
ns
****
****
Wnt3
Wnt3 + ephrinB2-WT
Wnt3 + ephrinB2-Y2F
Wnt3 + ephrinB2-Y2E
Control
d
Relative Siamois expression Normalized by ODC
1.5
1.0
0.5
0.0
****
ns
****
****
Wnt3
Wnt3+ephrinB2-WT
Wnt3+ephrinB2-Y2F
Wnt+ephrinB2-Y2E
Control
e
ephrinB2-HA
Wnt1-myc
Wnt3-myc
Wnt4-myc
Wnt5a-myc
Wnt8-myc
Wnt11-myc
caFGFR1
Uninjected
ephrinB2-HA
IP : ephrinB2 (HA)
Total lysate
p-Y300
p-Y312
p-Y326
ephrinB2(HA)
Wnts(myc)
Erk2
45
60
f
ephrinB2-V5
Ror2-HA
Ror2-HA + Wnt4-myc
caFGFR1
Uninjected
ephrinB2-V5
IP : ephrinB2 (V5)
Total lysate
p-Y(4G10)
ephrinB2(HA)
Wnt4(myc)
Ror2(HA)
ephrinB2(V5)
Erk2
45
60
100

Supplementary Fig. 4. ephrinB2 antagonizes Wnt/β-catenin signaling via interaction with PBM of Dsh2.

(a) Immunostaining for exogenously expressed GFP-β-catenin and ephrinB2-HA in ectodermal explants. Yellow arrowheads indicate the nuclear staining of β-catenin. Data means SEM. Numbers of neural plates/experiments: 9/3 for each column. One-way ANOVAs followed by Dunnett's multiple comparison tests were used. n.s., not significant, ****$p < 0.0001$. Scale bar, 20μm. (b) Co-IP assay with myc-Dsh2 and ephrinB2-WT-HA, ephrinB2-Y2E-HA or ephrinB2-Y2F. (c) Axis duplication assay along with *in situ* hybridization of the neuronal cell marker, *Sox2*. RNAs were injected into one side of the ventral marginal zone (VMZ) in the 4 cell stage embryos. Red arrowheads indicate the secondary axis. Immunoblotting represents the expression level of Wnt3 (0.5 pg) and ephrinB2 (100 pg). Data means SEM. Numbers of embryos/experiments: 74/3, 71/3, 73/3, 77/3, and 80/3 from left to right for each column. One-way ANOVAs followed by Dunnett's multiple comparison tests were used. n.s., not significant, ****$p < 0.0001$. Scale bar, 500μm. (d) *Siamois* expression levels by Quantitative RT-PCR assay in ectodermal explants at stage 10. (e) Level of phospho-Tyrosine in ephrinB2 by Immunoblots using specific phospho-tyrosine Abs (pY300, pY312, or pY326) at stage 13 embryos. (f) Level of phospho-Tyrosine in ephrinB2 by Immunoblots using pan-phospho-tyrosine antibodies (4G10).

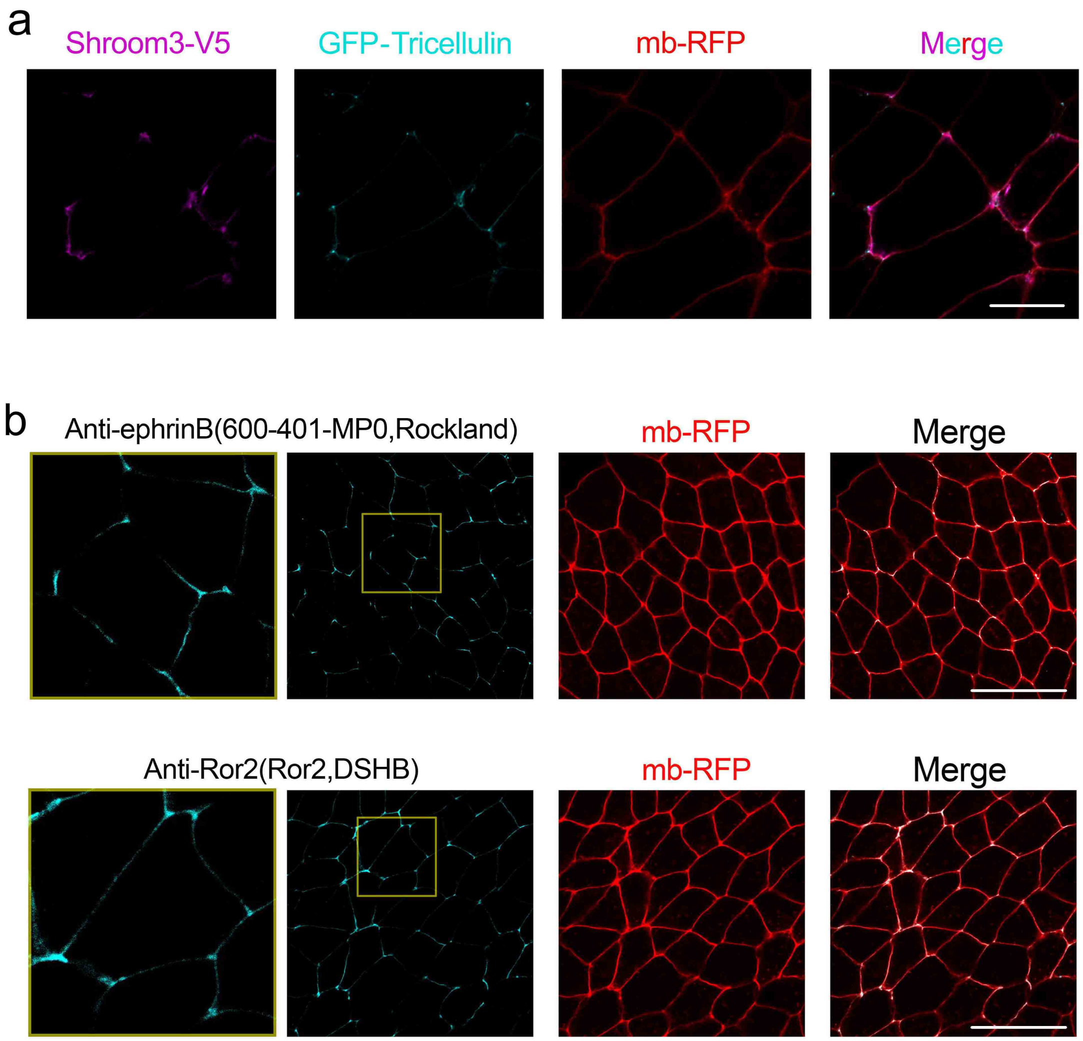
a
Shroom3-V5
GFP-Tricellulin
mb-RFP
Merge
b
Anti-ephrinB(600-401-MP0,Rockland)
mb-RFP
Merge
Anti-Ror2(Ror2,DSHB)
mb-RFP
Merge

Supplementary Fig. 5. WERDS localizes at tricellular junctions during apical constriction. (a) Representative images of Shroom3 (Magenta) subcellular localization in neural plate cells of Stage 15 embryos. Exogenously expressed GFP-Tricellulin (Cyan) marked the Tricellular junctions. Membrane-RFP labeled cell boundaries. Scale bar, 10μm. (b) Representative images of endogenous ephrinB2 or Ror2 subcellular localization in neural plate cells of Stage 15 embryos. Membrane-RFP labeled cell boundaries. Scale bar, 50μm.

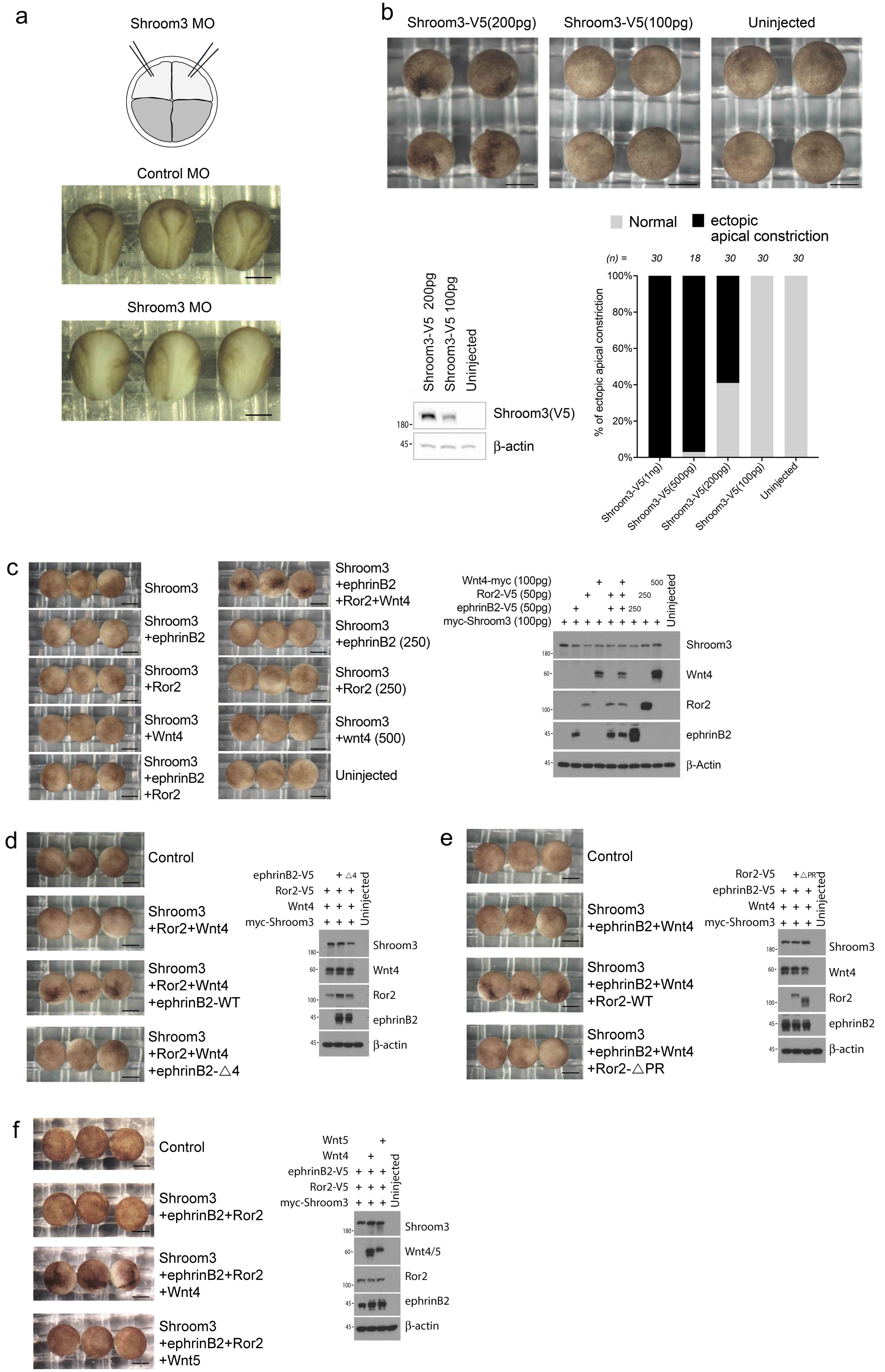

a
Shroom3 MO
Control MO
Shroom3 MO
b
Shroom3-V5(200pg)
Shroom3-V5(100pg)
Uninjected
Normal
ectopic apical constriction
(n) = 30 18 30 30 30
% of ectopic apical constriction
Shroom3-V5(1ng)
Shroom3-V5(500pg)
Shroom3-V5(200pg)
Shroom3-V5(100pg)
Uninjected
Shroom3-V5 200pg
Shroom3-V5 100pg
Uninjected
Shroom3(V5)
β-actin
c
Shroom3
Shroom3 +ephrinB2
Shroom3 +Ror2
Shroom3 +Wnt4
Shroom3 +ephrinB2 +Ror2
Shroom3 +ephrinB2 +Ror2+Wnt4
Shroom3 +ephrinB2 (250)
Shroom3 +Ror2 (250)
Shroom3 +wnt4 (500)
Uninjected
Wnt4-myc (100pg)
Ror2-V5 (50pg)
ephrinB2-V5 (50pg)
myc-Shroom3 (100pg)
Shroom3
Wnt4
Ror2
ephrinB2
β-Actin
d
Control
Shroom3 +Ror2+Wnt4
Shroom3 +Ror2+Wnt4 +ephrinB2-WT
Shroom3 +Ror2+Wnt4 +ephrinB2-△4
ephrinB2-V5
Ror2-V5
Wnt4
myc-Shroom3
Uninjected
Shroom3
Wnt4
Ror2
ephrinB2
β-actin
e
Control
Shroom3 +ephrinB2+Wnt4
Shroom3 +ephrinB2+Wnt4 +Ror2-WT
Shroom3 +ephrinB2+Wnt4 +Ror2-△PR
Ror2-V5
ephrinB2-V5
Wnt4
myc-Shroom3
Uninjected
Shroom3
Wnt4
Ror2
ephrinB2
β-actin
f
Control
Shroom3 +ephrinB2+Ror2
Shroom3 +ephrinB2+Ror2 +Wnt4
Shroom3 +ephrinB2+Ror2 +Wnt5
Wnt5
Wnt4
ephrinB2-V5
Ror2-V5
myc-Shroom3
Uninjected
Shroom3
Wnt4/5
Ror2
ephrinB2
β-actin

Supplementary Fig. 6. WERDS signaling complex induces ectopic apical constriction.

(a) Representative embryo phenotypes of Shroom3 MO knockdown. MOs were injected D1.1 into two blastomeres of the 8-cell stage embryos. The image was taken at stage 16. Scale bar, 500µm. (b) Representative images of ectopic apical constriction induced by Shroom3. Immunoblotting represents the expression level of Shroom3. The histogram depicts the quantification of the phenotypes. Scale bar, 500µm. (c) Representative images of ectopic apical constriction by exogenously expressed WERDS complex. Immunoblotting represents the expression level of WERDS components. Scale bar, 500µm. (d) Ectopic apical constriction analysis with the WERDS complex and ephrinB2-Δ4. Scale bar, 500µm. (e) Ectopic apical constriction analysis with the WERDS complex and Ror2-ΔPR. Scale bar, 500µm. (f) Ectopic apical constriction analysis with the WERDS complex and Wnt5a. Scale bar, 500µm.
