# Supplementary material for: Wnt4 and ephrinB2 instruct apical constriction via Dishevelled and non-canonical signaling
Source: Nat Commun. 2023 Jan 20;14:337. doi: 10.1038/s41467-023-35991-6 (PMC9860048; doi:10.1038/s41467-023-35991-6)
Supplement: Supplementary file 3 — Description of Additional Supplementary Files [file 41467_2023_35991_MOESM3_ESM.pdf]

Description of Additional Supplementary Files

File Name: Supplementary Data 1
Description: **Raw mass spectrometry results from ephrinB2 pull-down from neural plates.**

File Name: Supplementary Data 2
Description: **Oligos information (MOs, sgRNA, and RT-PCR primers)**

File Name: Supplementary Movie 1
Description: **Neural tube closure defects in ephrinB2, Ror2, or Wnt4 morphants.** Time-lapse images of Xenopus embryos from stage 13 to 20. MOs were injected into the D1.1 blastomere at the 16 cell stage embryos. Scale bar, 500mm. One frame per 10 min.

File Name: Supplementary Movie 2
Description: **Neural tube closure defects in ephrinB2 or Ror2 knockout embryos.** Time-lapse images of Xenopus embryos from stage 13 to 20. sgRNA and Cas9 proteins were injected at the one-cell stage embryos. Scale bar, 500mm. One frame per 10 min.

File Name: Supplementary Movie 3
Description: **Contractile actin bundle formation in ephrinB2 morphants.** Time-lapse confocal images of neural plate cells of ephrinB2 morphants. MOs and RNAs along with GFP-UtrCH mRNA were injected into the D1.1 blastomere at the 16 cell stage embryos. Dotted line boxes indicate the magnified area. The red asterisk indicates V1.1 blastomere which becomes non-neural ectoderm. Scale bar, 50mm. One frame per 15 seconds.

File Name: Supplementary Movie 4
Description: **Contractile actin bundle formation in Ror2 morphants.** Time-lapse confocal images of neural plate cells of Ror2 morphants. MOs and RNAs along with GFP-UtrCH mRNA were injected into the D1.1 blastomere at the 16 cell stage embryos. Dotted line boxes indicate the magnified area. Scale bar, 50mm. One frame per 15 seconds.

File Name: Supplementary Movie 5
Description: **Neural tube closure defects in Wnt4 knockout embryos.** Time-lapse images of *Xenopus* embryos from stage 13 to 20. sgRNA and Cas9 proteins were injected at the one-cell stage embryos. Scale bar, 500mm. One frame per 10 min.

File Name: Supplementary Movie 6
Description: **Contractile actin bundle formation in Wnt4 morphants.** Time-lapse confocal images of neural plate cells of Wnt4 morphants. MOs and RNAs along with GFP-UtrCH mRNA were injected into the D1.1 blastomere at the 16 cell stage embryos. Dotted line boxes indicate the magnified area. Scale bar, 50mm. One frame per 15 seconds.
