# Supplementary material for: Wnt4 and ephrinB2 instruct apical constriction via Dishevelled and non-canonical signaling
Source: Nat Commun. 2023 Jan 20;14:337. doi: 10.1038/s41467-023-35991-6 (PMC9860048; doi:10.1038/s41467-023-35991-6)
Supplement: Supplementary file 12 — Reporting Summary [file 41467_2023_35991_MOESM12_ESM.pdf]

nature portfolio

Corresponding author(s): Ira O. Daar & Jaeho Yoon

Last updated by author(s): Nov 22, 2022

Reporting Summary

Nature Portfolio wishes to improve the reproducibility of the work that we publish. This form provides structure for consistency and transparency in reporting. For further information on Nature Portfolio policies, see our Editorial Policies and the Editorial Policy Checklist.

Statistics

For all statistical analyses, confirm that the following items are present in the figure legend, table legend, main text, or Methods section.

n/a	Confirmed	
☐	☒	The exact sample size (*n*) for each experimental group/condition, given as a discrete number and unit of measurement
☐	☒	A statement on whether measurements were taken from distinct samples or whether the same sample was measured repeatedly
☐	☒	The statistical test(s) used AND whether they are one- or two-sided<br>*Only common tests should be described solely by name; describe more complex techniques in the Methods section.*
☐	☒	A description of all covariates tested
☐	☒	A description of any assumptions or corrections, such as tests of normality and adjustment for multiple comparisons
☐	☒	A full description of the statistical parameters including central tendency (e.g. means) or other basic estimates (e.g. regression coefficient) AND variation (e.g. standard deviation) or associated estimates of uncertainty (e.g. confidence intervals)
☐	☒	For null hypothesis testing, the test statistic (e.g. *F*, *t*, *r*) with confidence intervals, effect sizes, degrees of freedom and *P* value noted<br>*Give P values as exact values whenever suitable.*
☒	☐	For Bayesian analysis, information on the choice of priors and Markov chain Monte Carlo settings
☐	☒	For hierarchical and complex designs, identification of the appropriate level for tests and full reporting of outcomes
☒	☐	Estimates of effect sizes (e.g. Cohen's *d*, Pearson's *r*), indicating how they were calculated

Our web collection on statistics for biologists contains articles on many of the points above.

Software and code

Policy information about availability of computer code

Data collection	CRISPRscan website (https://www.crisprscan.org) for the guide RNA design.
Data analysis	Data were analyzed with Fiji (version: 2.9.0). Prism9 (version: 9.4.1) program was used for all statistical analysis. No custom algorithms or codes were used in this study.

For manuscripts utilizing custom algorithms or software that are central to the research but not yet described in published literature, software must be made available to editors and reviewers. We strongly encourage code deposition in a community repository (e.g. GitHub). See the Nature Portfolio guidelines for submitting code & software for further information.

Data

Policy information about availability of data

All manuscripts must include a data availability statement. This statement should provide the following information, where applicable:

- Accession codes, unique identifiers, or web links for publicly available datasets
- A description of any restrictions on data availability
- For clinical datasets or third party data, please ensure that the statement adheres to our policy

Raw mass spectrometry Data are supplied as Supplementary Data file and all source data presented in graphs within the Figures are provided as a Source Data file. Additional information is available from the corresponding authors J.Y. (jaeho.yoon@nih.gov) upon request.

Human research participants

Policy information about studies involving human research participants and Sex and Gender in Research.

Reporting on sex and gender	No human research samples were used in this study.
Population characteristics	No human research samples were used in this study.
Recruitment	No human research samples were used in this study.
Ethics oversight	No human research samples were used in this study.

Note that full information on the approval of the study protocol must also be provided in the manuscript.

Field-specific reporting

Please select the one below that is the best fit for your research. If you are not sure, read the appropriate sections before making your selection.

☒ Life sciences ☐ Behavioural & social sciences ☐ Ecological, evolutionary & environmental sciences

For a reference copy of the document with all sections, see nature.com/documents/nr-reporting-summary-flat.pdf

Life sciences study design

All studies must disclose on these points even when the disclosure is negative.

Sample size	No statistical methods were used to predetermine the sample size. All experiments include at least three biological replicates for the analysis of statistical significance, and all sample sizes are reported in the Figure legends. Stated in the Material and Methods, Statistical analysis. Dead cells and embryos were excluded from all experimental analysis. Embryos which have mis-targeted injection also were excluded (the target injection was confirmed by co-injection of lineage tracer (GFP RNAs, green or red fluorescent Dextran)).
Data exclusions	No data was excluded.
Replication	All experiments include at least three independent biological replicates. All experiments were performed and statistical analysis done independently of these data sets.
Randomization	Embryos were selected for control or manipulated groups randomly. No specific experimental group were defined and all data were considered.
Blinding	Embryos were analyzed blindly. We have not used any specific sampling nor we had exclude any data.

Reporting for specific materials, systems and methods

We require information from authors about some types of materials, experimental systems and methods used in many studies. Here, indicate whether each material, system or method listed is relevant to your study. If you are not sure if a list item applies to your research, read the appropriate section before selecting a response.

Materials & experimental systems

n/a	Involved in the study	
☐	☒	Antibodies
☒	☐	Eukaryotic cell lines
☒	☐	Palaeontology and archaeology
☐	☒	Animals and other organisms
☒	☐	Clinical data
☒	☐	Dual use research of concern

Methods

n/a	Involved in the study	
☒	☐	ChIP-seq
☒	☐	Flow cytometry
☒	☐	MRI-based neuroimaging

Antibodies

Antibodies used	Rabbit polyclonal anti-ephrinB type ligands (600-401-MP0, 1:2000), Rockland<br>Mouse monoclonal anti-Ror2 (Ror2, 1:200), Developmental Studies Hybridoma Bank<br>Rabbit polyclonal anti-phospho-MLC (ab2480, 1:500), Abcam

	Goat polyclonal anti-GFP-Perdoxidase (600-103-215 , 1:2000), Rockland<br>Rabbit polyclonal anti-Erk2 (C-14) (sc-154, 1:1000), Santacruz Biotechnology<br>Mouse monoclonal anti-b-Actin (sc-47778 : 1:1000), Santacruz Biotechnology<br>Anti-Phosphotyrosine Antibody (clone 4G10) (05-321, 1:1000), Millipore Sigma<br>Anti-EphrinB (Tyr298) Antibody (p1110-298, 1:1000), PhosphoSolutions<br>Anti-EphrinB (Tyr317) Antibody (p1110-317, 1:1000), PhosphoSolutions<br>Anti-EphrinB (Tyr331) Antibody (p1110-331, 1:1000), PhosphoSolutions<br>Rabbit polyclonal anti-GFP Alexa FluorTM conjugated-488 (A-21311, 1:500), Thermo Fisher Scientific<br>Rabbit polyclonal anti-RFP CF® dye conjugated-594 (20422, 1:500), Biotium<br>Alexa Fluor 488-conjugated Goat anti-rabbit (A11034, 1:500), Thermo Fisher Scientific<br>Alexa Fluor 594-conjugated Goat anti-mouse (A11005, 1:500), Thermo Fisher Scientific<br>Goat Anti-Mouse IgG Antibody, HRP conjugate (12-349, 1:1000), Millipore Sigma<br>Goat Anti-Rabbit IgG Antibody, HRP-conjugate (12-348, 1:1000), Millipore Sigma<br>Rat monoclonal anti-HA-Peroxidase (clone 3F10) (12013819001, 1:1000), Roche<br>Mouse monoclonal anti-FLAG M2-Peroxidase (A8592, 1:1000), Sigma-Aldrich<br>Mouse monoclonal anti-Myc-Peroxidase (16-212, 1:1000), Millipore Sigma<br>Mouse monoclonal anti-V5-Peroxidase (V2260, 1:1000), Millipore Sigma<br>ANTI-FLAG M2 Affinity Gel (A2220, 25ul for sample), Millipore Sigma<br>HA Epitope Tag Antibody Agarose conjugate (sc-500777, 25ul for sample), Santacruz Biotechnology<br>V5-Trap agarose (v5ta, 25 ul for sample), Chemotek<br>GFP-Trap agarose (gta-100, 25ul for sample), Chemotek
Validation	These are standard antibodies validated in a host of previous publications.

Animals and other research organisms

Policy information about studies involving animals; ARRIVE guidelines recommended for reporting animal research, and Sex and Gender in Research

Laboratory animals	Adults female Xenopus laevies (from NASCO, 1.5 year old), Adults male Xenopus laevies (from NASCO, 2 year old)Adults female Xenopus laevies were used to obtain oocytes. Adults male Xenopus laevies were used to obtain sperm.
Wild animals	None
Reporting on sex	All experiments were concluded before sex determination. Sex is genetically defined in Xenopus with 50:50 ratios.
Field-collected samples	No field collected samples were used in the study
Ethics oversight	All animal studies were approved by the NCI at Frederick Animal Care and Use Committee [ACUC].

Note that full information on the approval of the study protocol must also be provided in the manuscript.
