# Supplementary material for: Wnt4 and ephrinB2 instruct apical constriction via Dishevelled and non-canonical signaling
Source: Nat Commun. 2023 Jan 20;14:337. doi: 10.1038/s41467-023-35991-6 (PMC9860048; doi:10.1038/s41467-023-35991-6)

Fig.1c

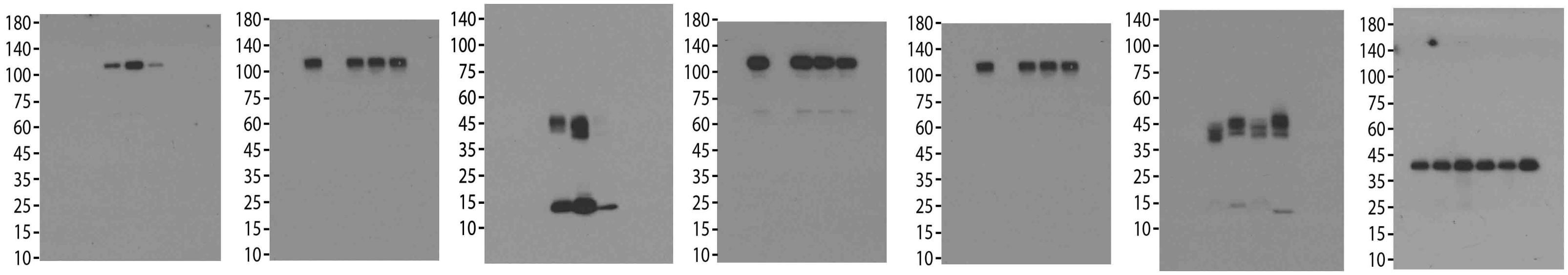

Fig.1f

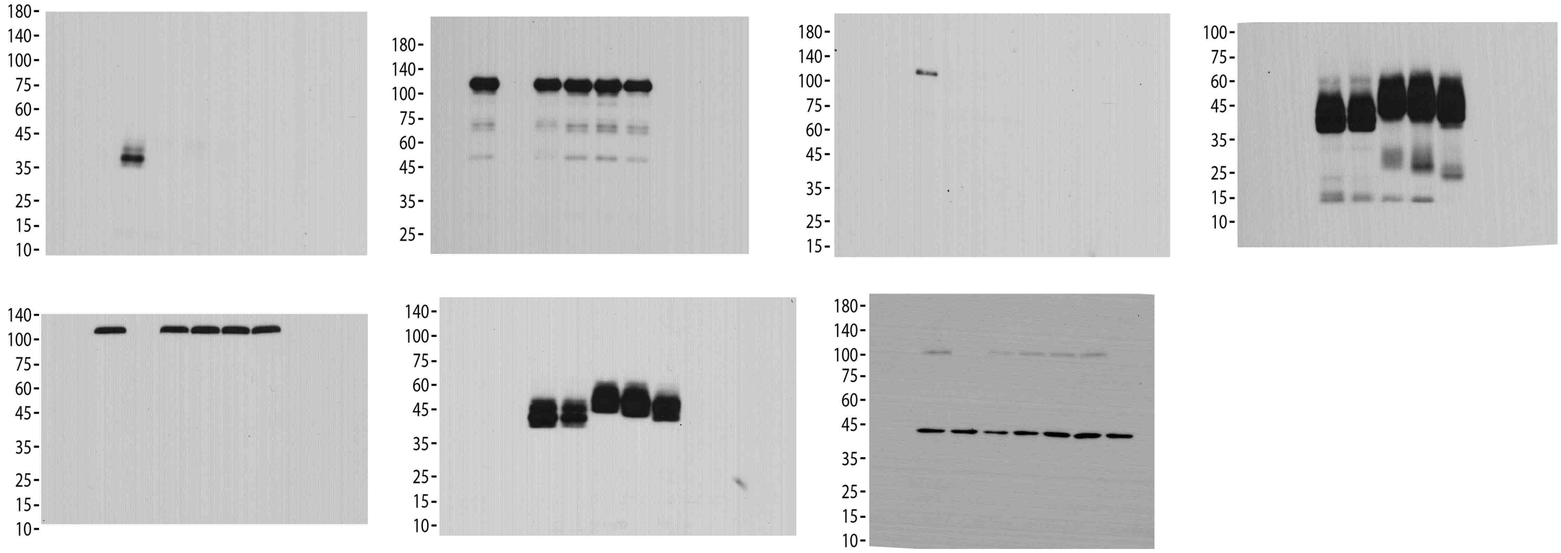

Fig.1g

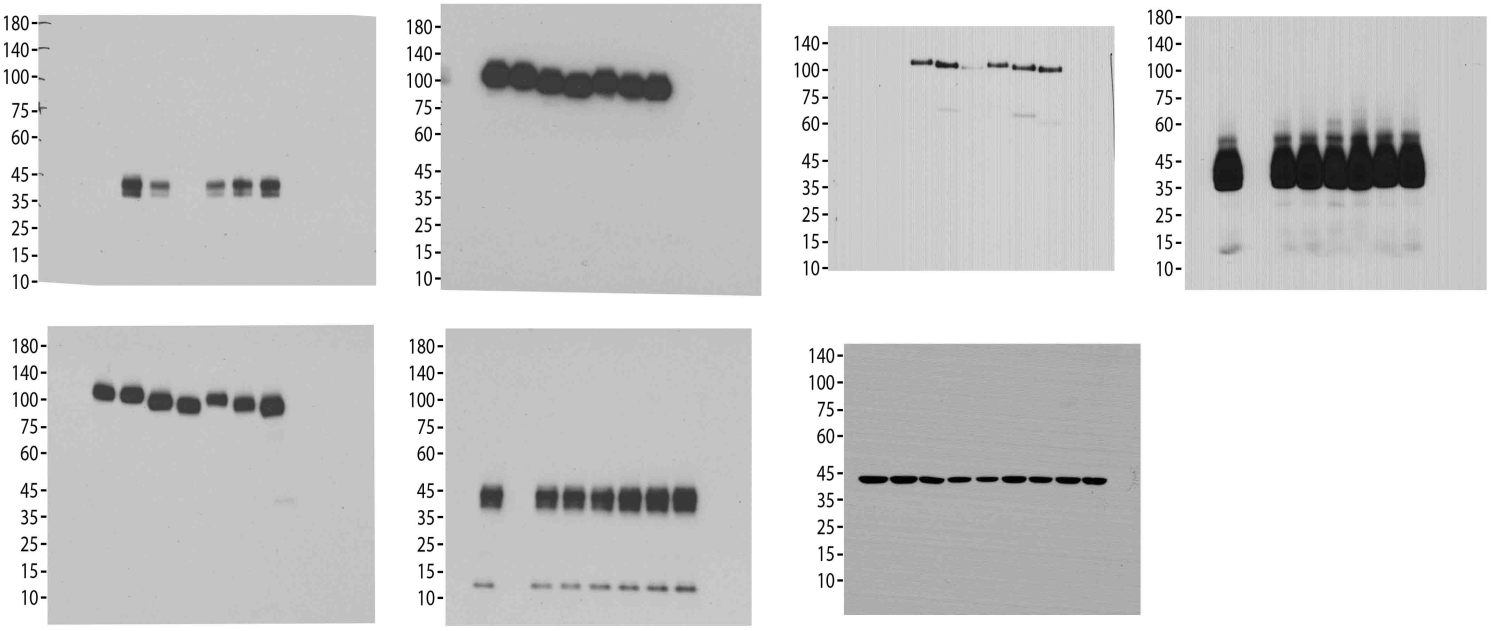

Fig.1h

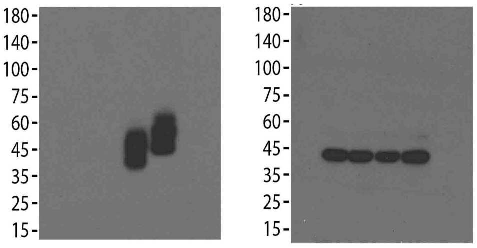

Fig.1i

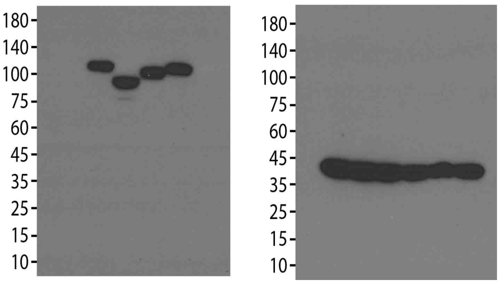

Fig. 3a

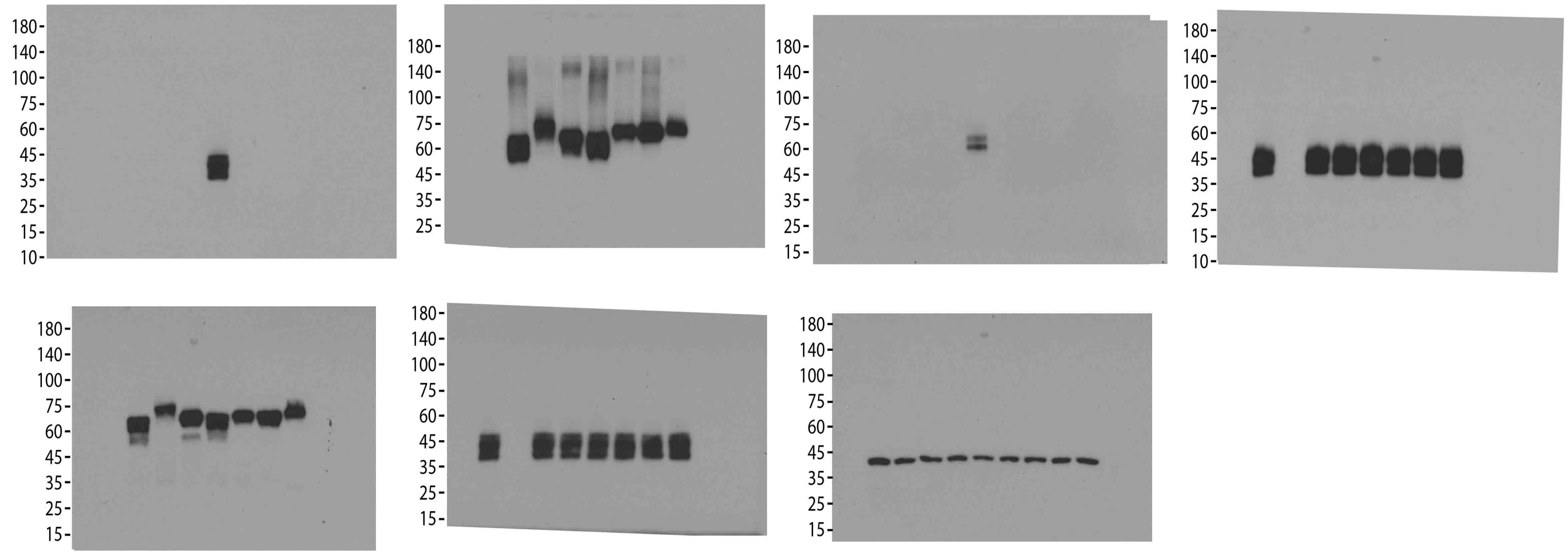

Fig. 3b

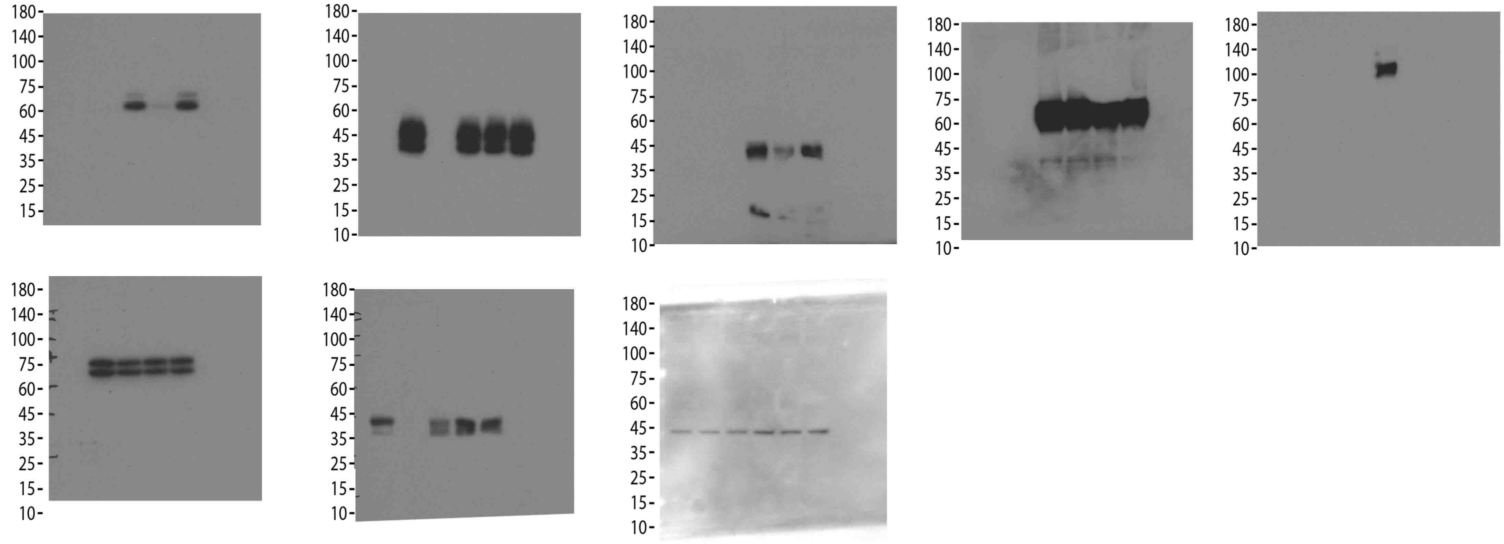

Fig. 3c

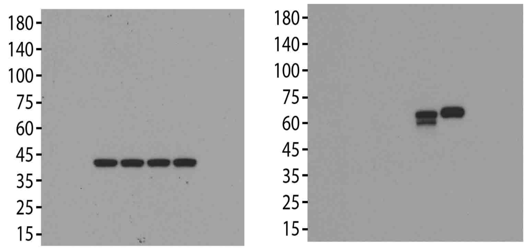

Fig. 4a

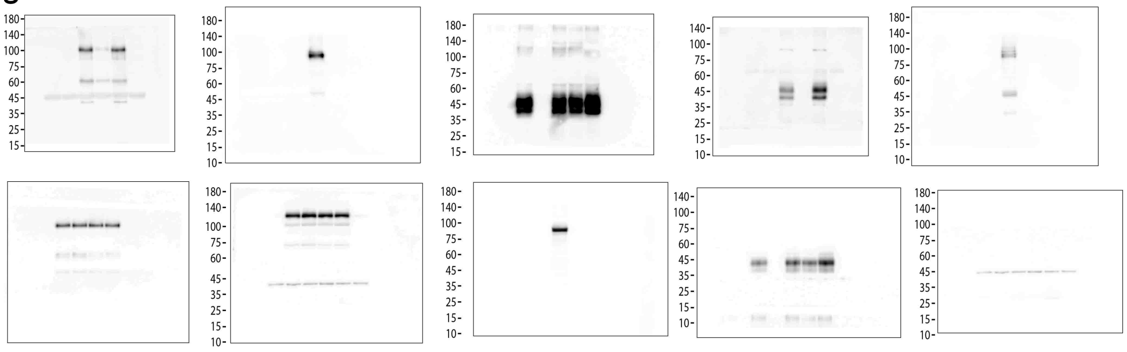

Fig. 4c

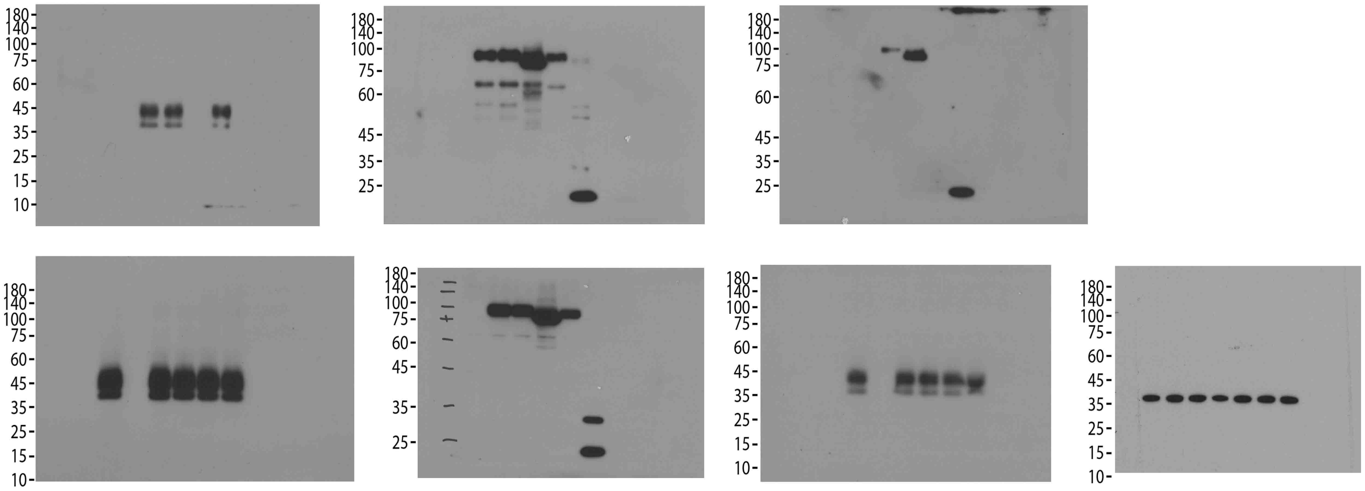

Fig. 4d

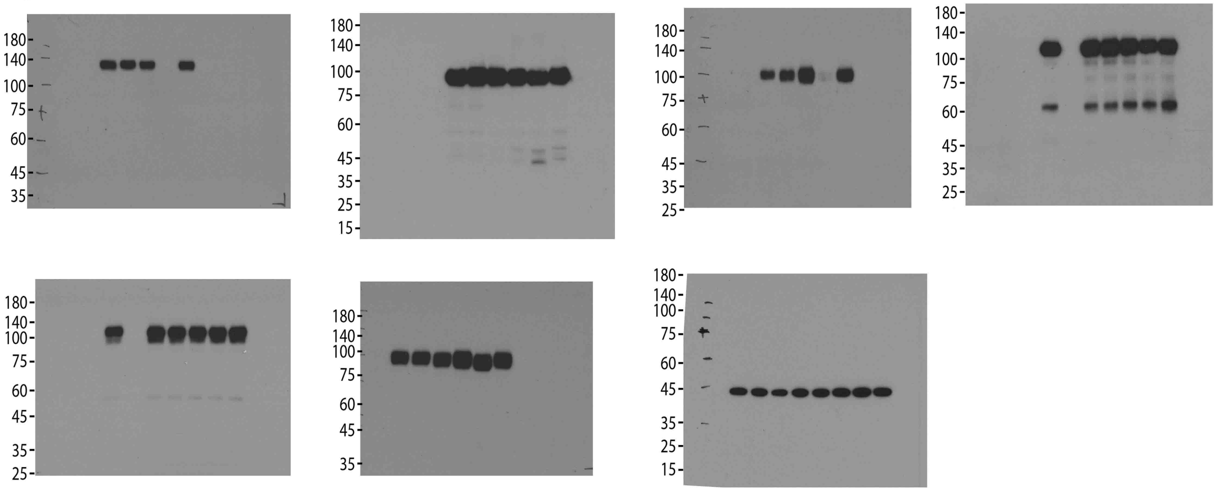

Fig. 4e

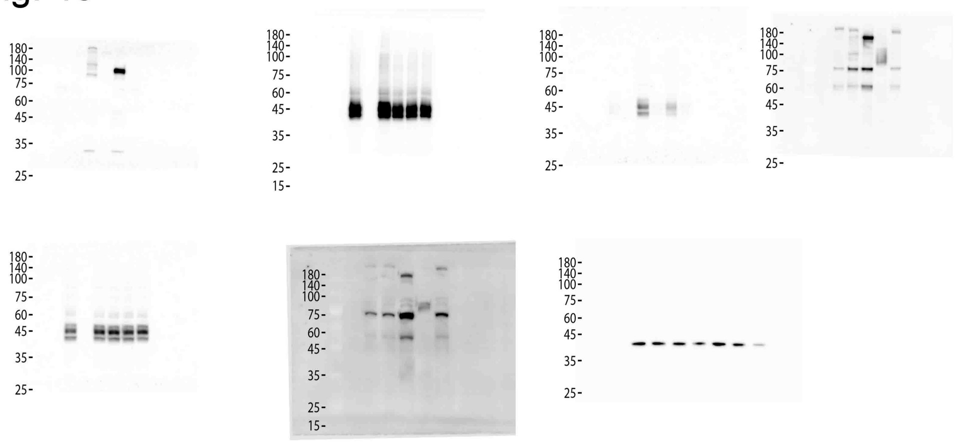

Fig. 4f

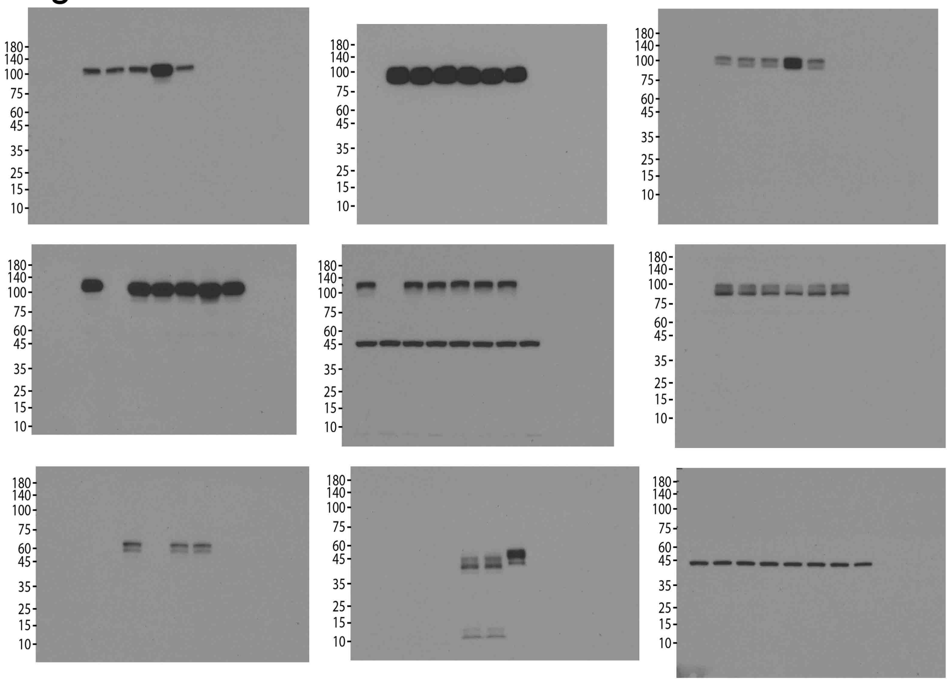

Fig. 4g

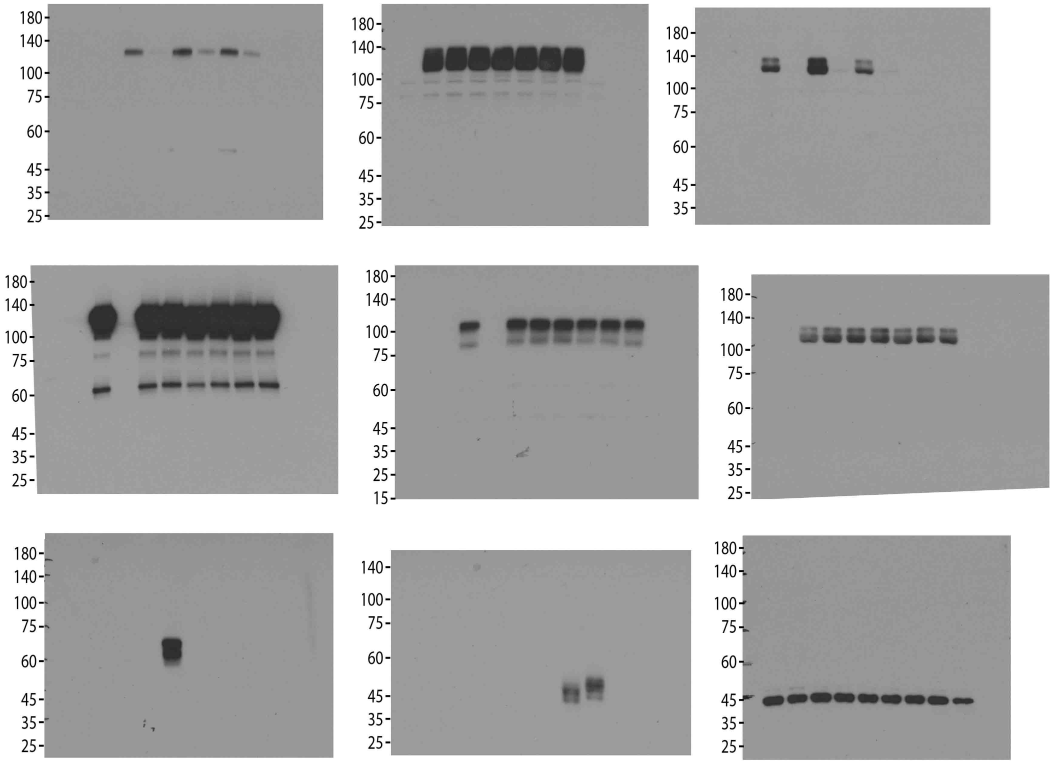

Fig. 4h

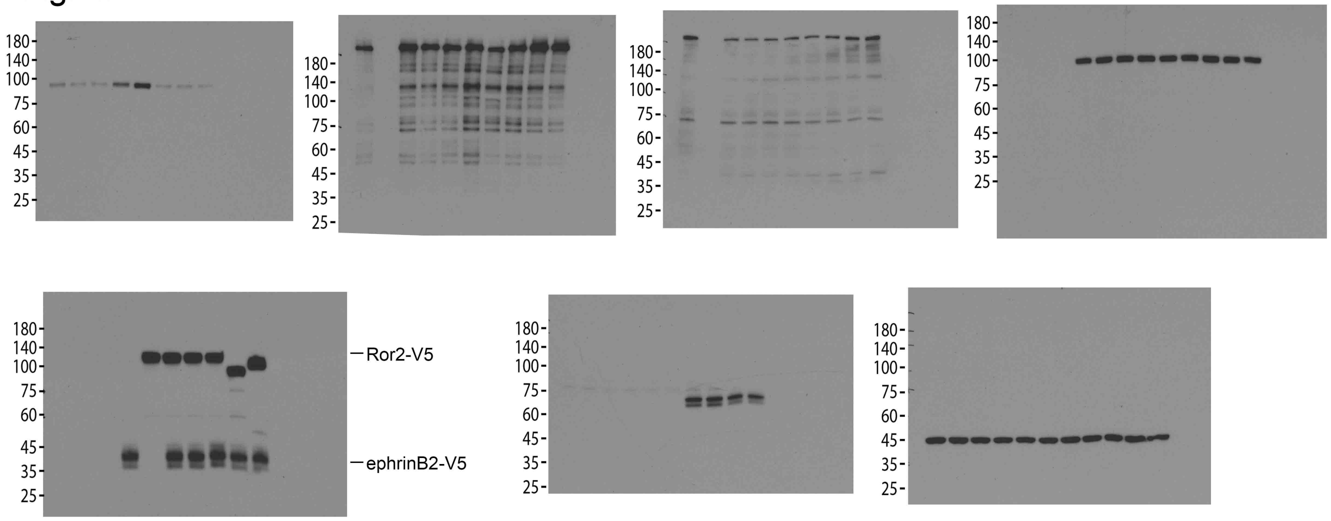
180
140
100
75
60
45
35
25
Ror2-V5
ephrinB2-V5

Fig. 4i

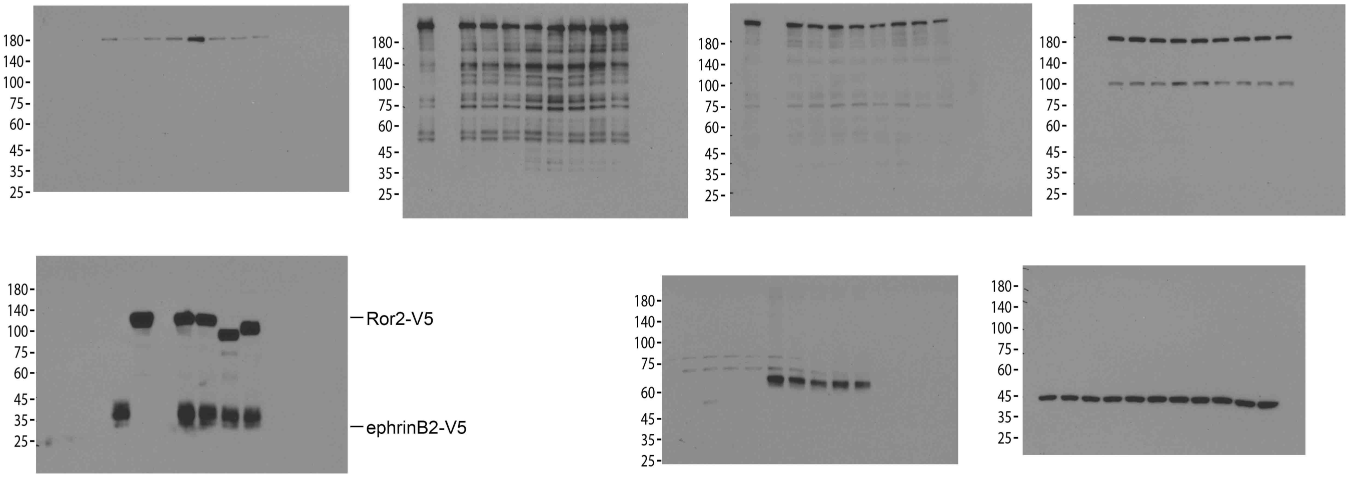
180
140
100
75
60
45
35
25
Ror2-V5
ephrinB2-V5

Supplementary Fig. 1b

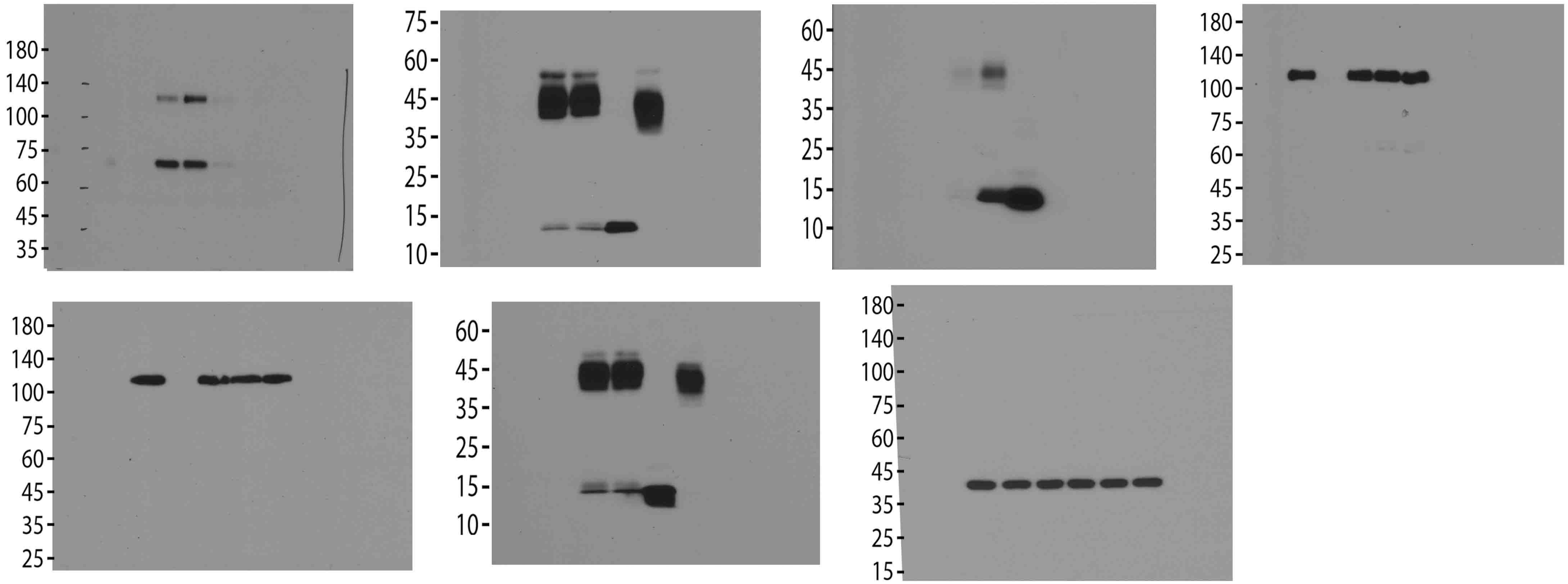

Supplementary Fig. 1d

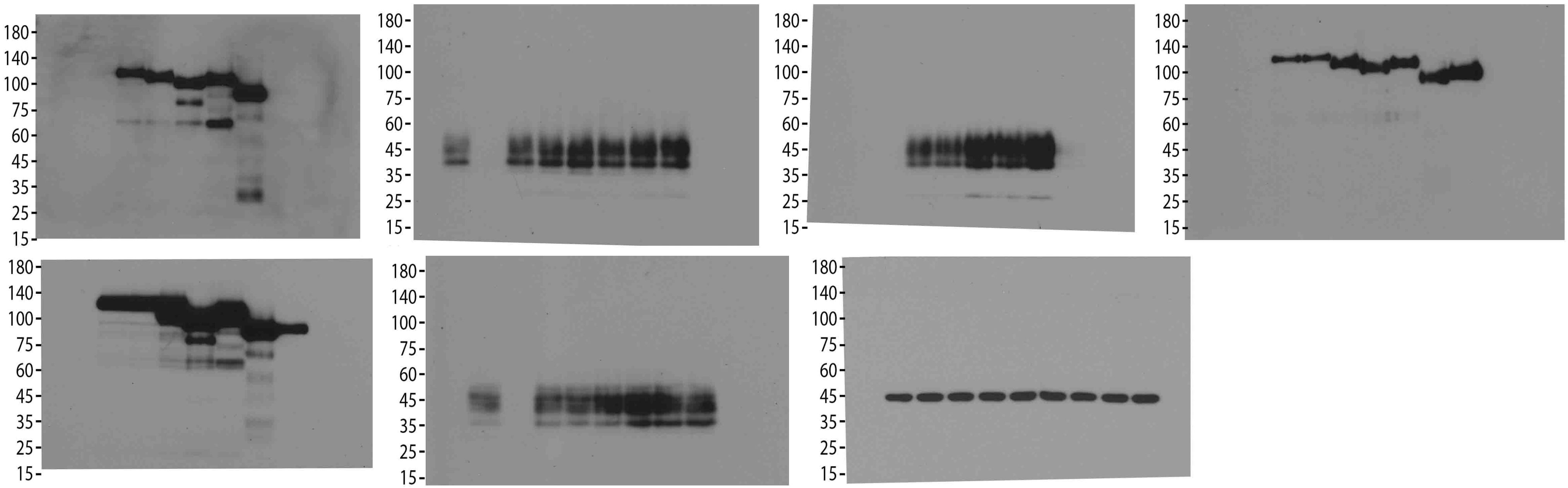

Supplementary Fig. 2a

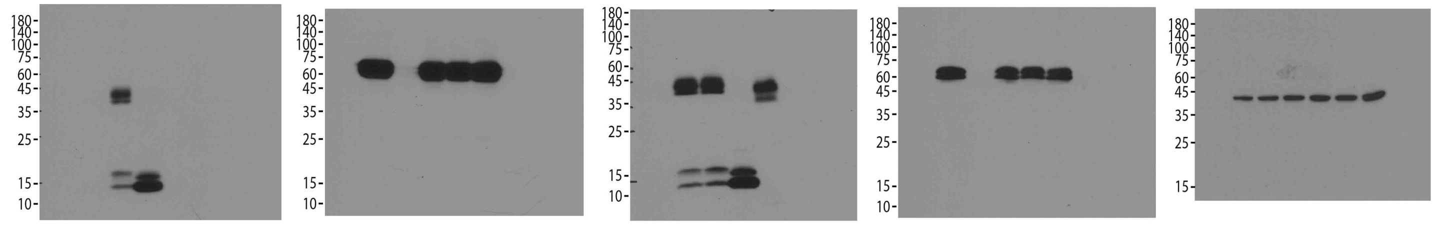

Supplementary Fig. 2b

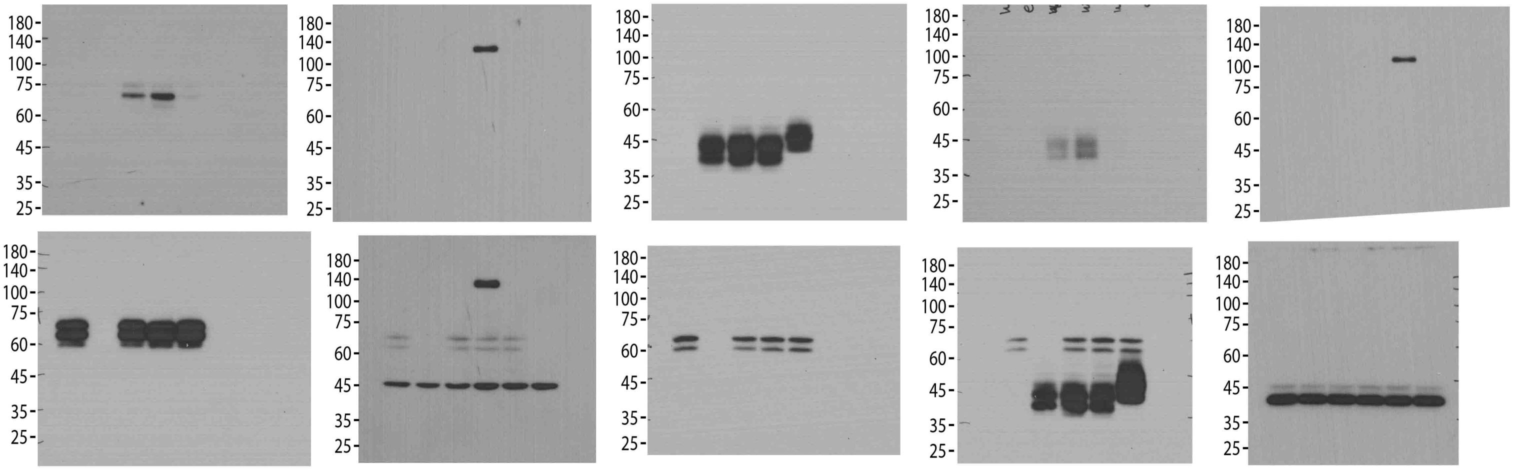

Supplementary Fig. 2c

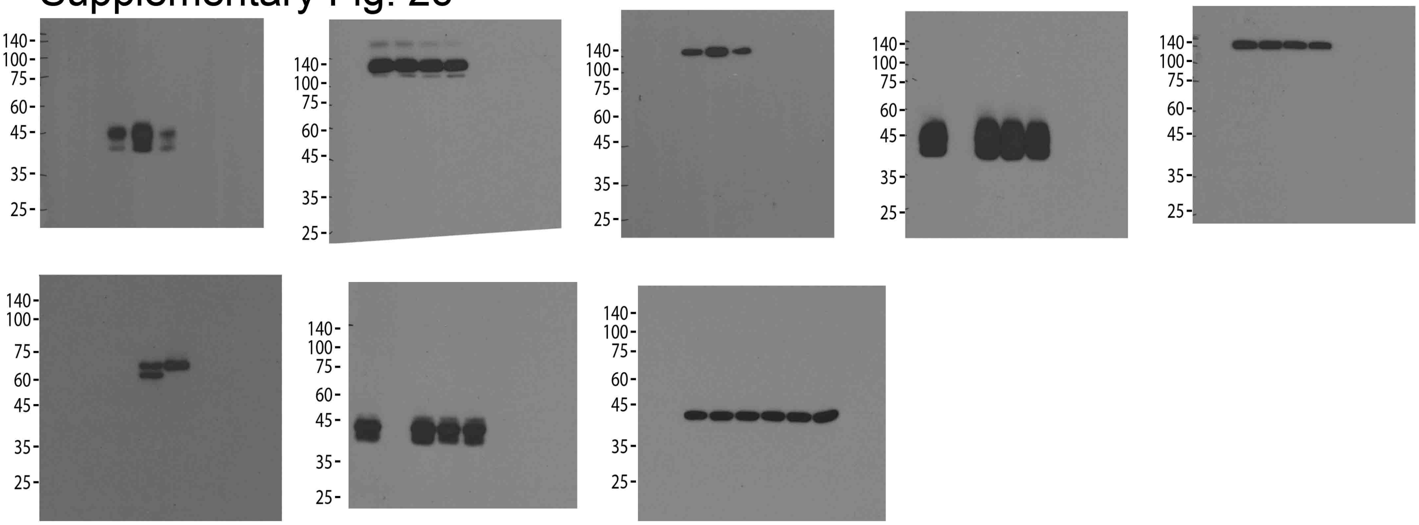

Supplementary Fig. 2d

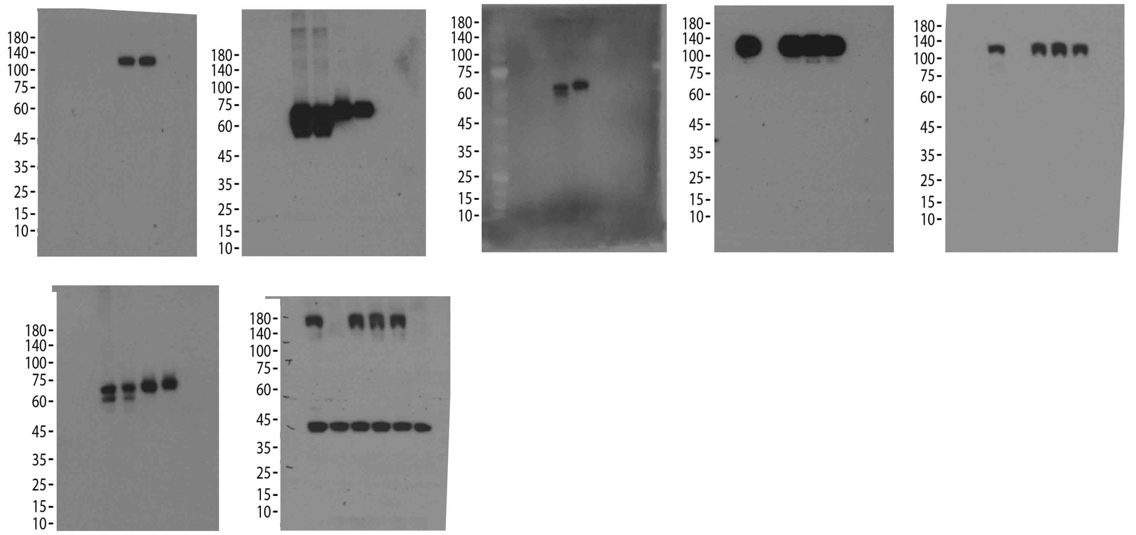

Supplementary Fig. 3a_1

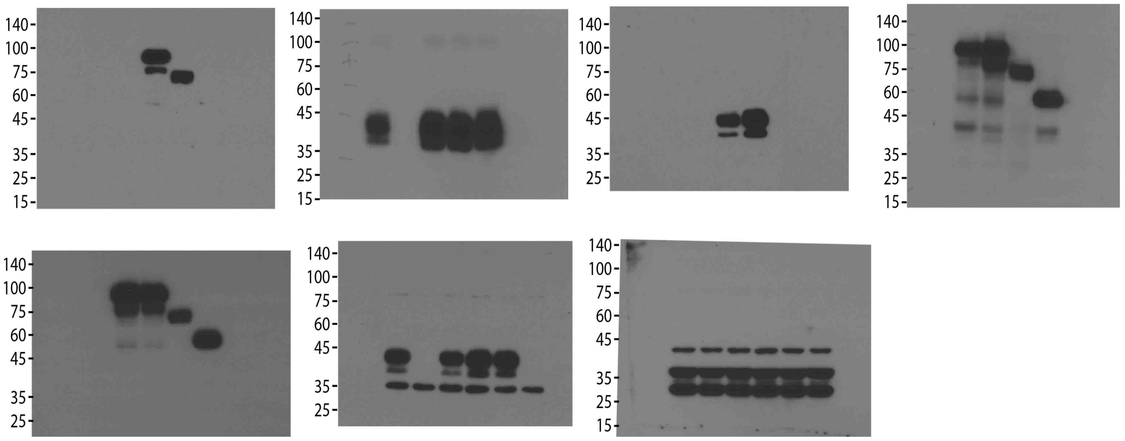

Supplementary Fig. 3a_2

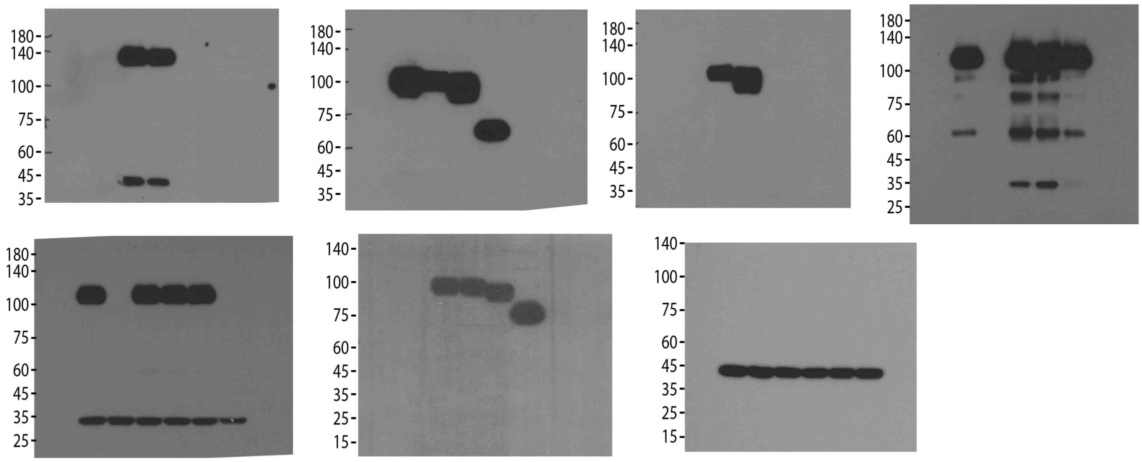

Supplementary Fig. 3b_1

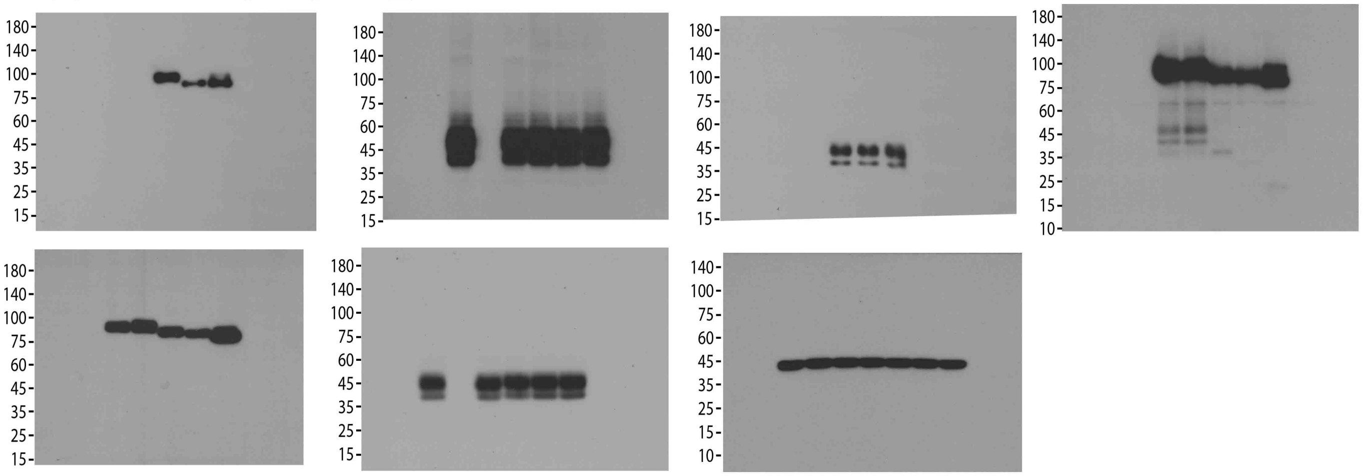

Supplementary Fig. 3b_2

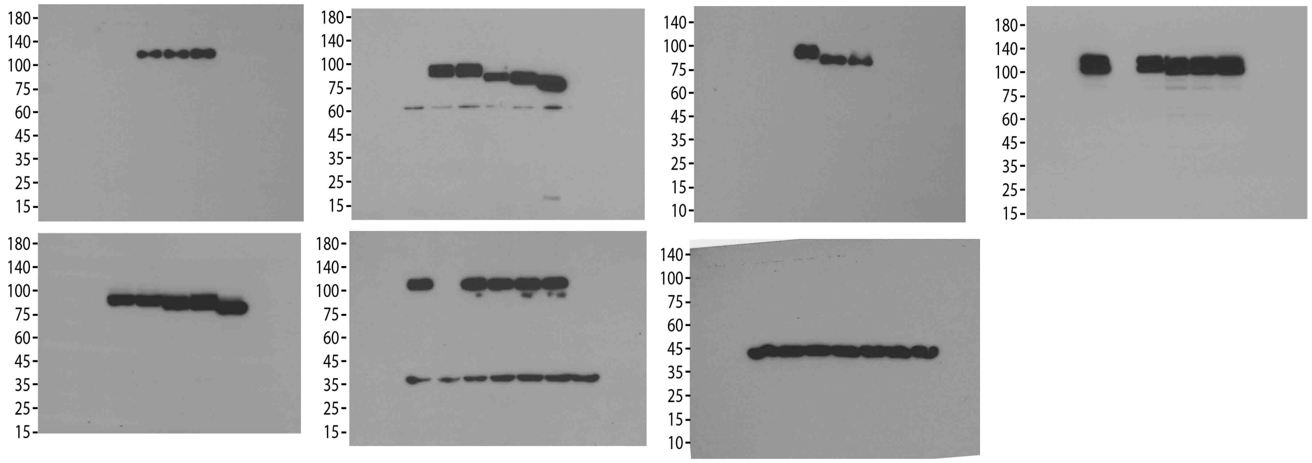

Supplementary Fig. 3c_1

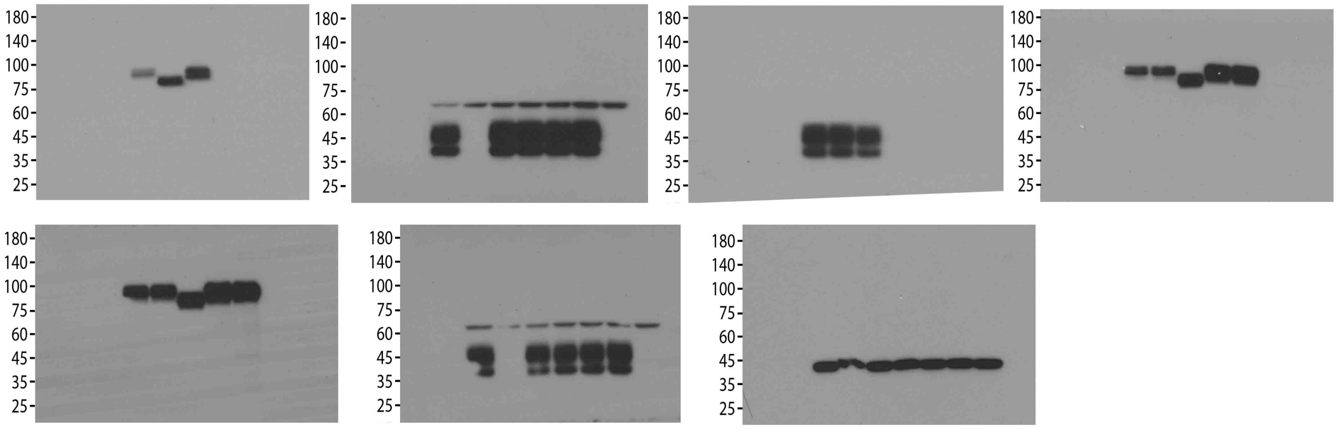

Supplementary Fig. 3c_2

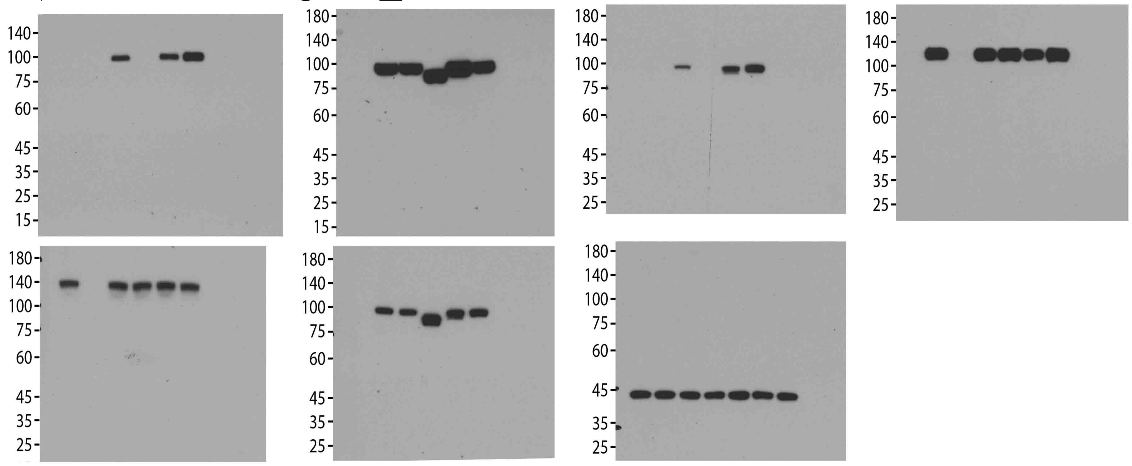

Supplementary Fig. 3d

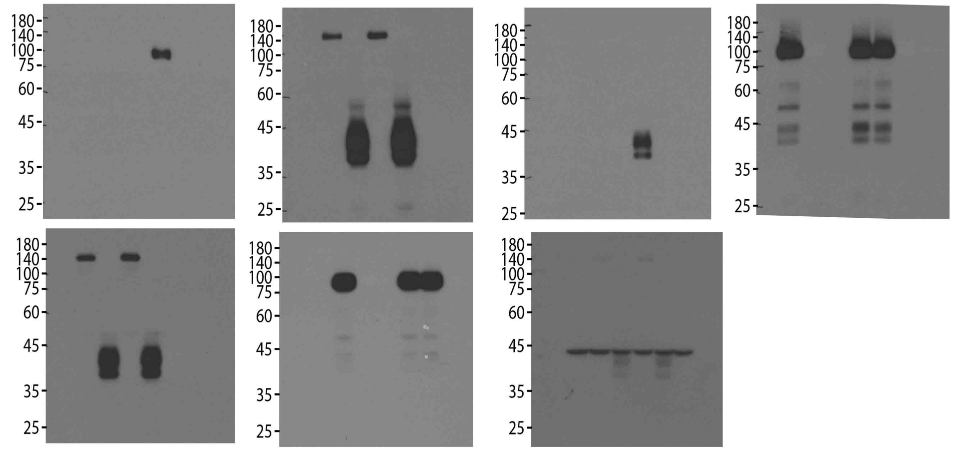

Supplementary Fig. 3e

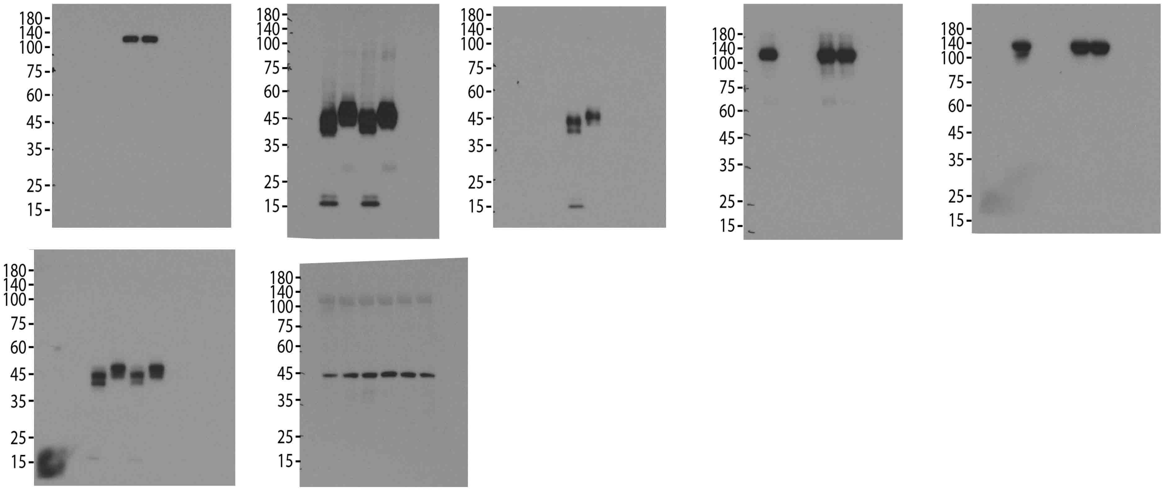

Supplementary Fig. 3f

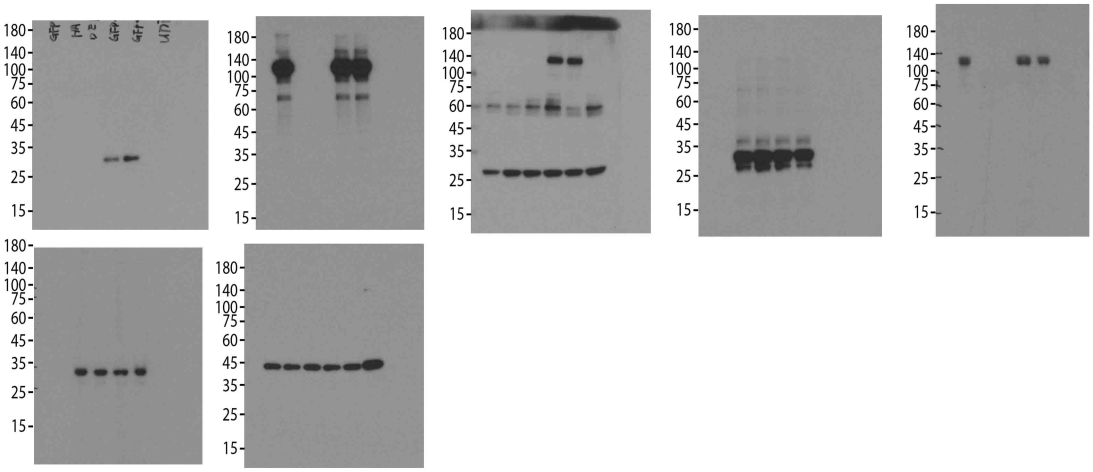

Supplementary Fig. 3g

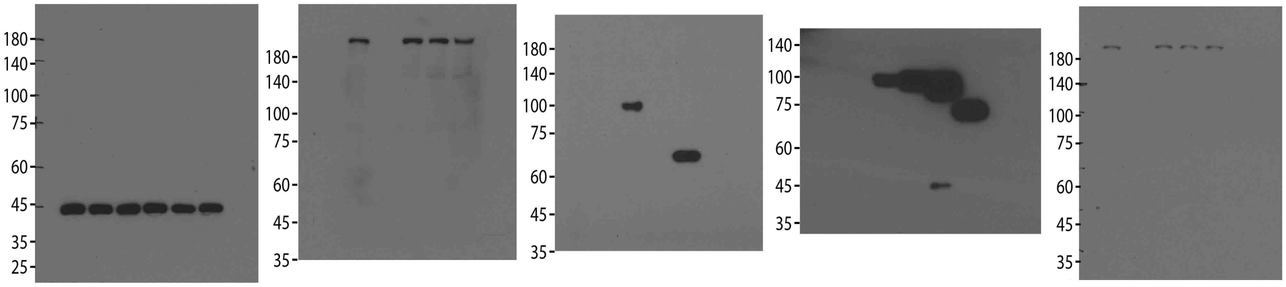

Supplementary Fig. 3j

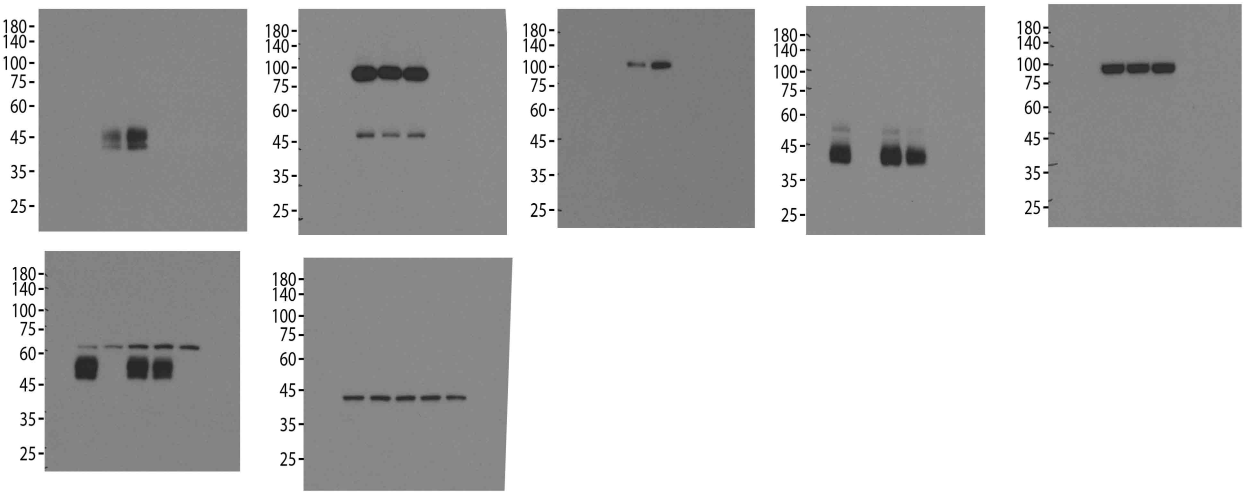

Supplementary Fig. 3k

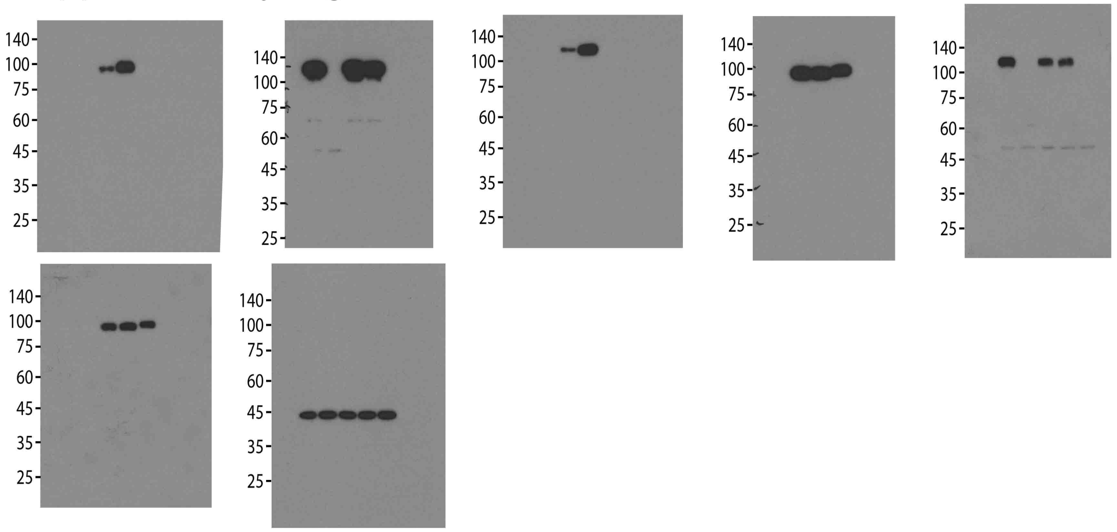

Supplementary Fig. 4b

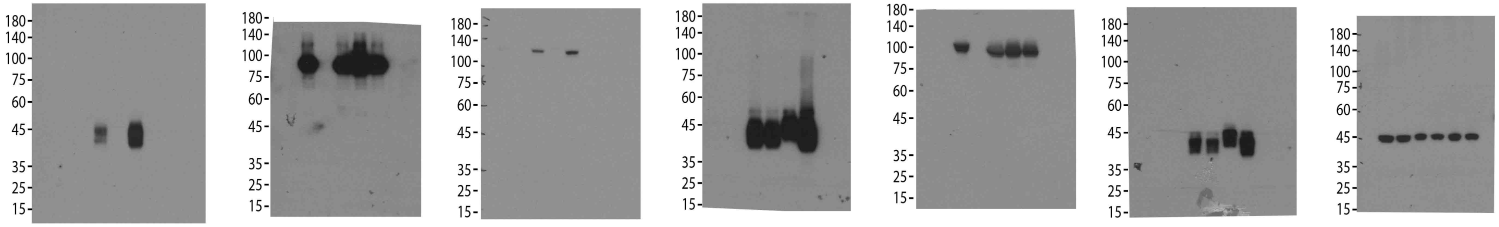

Supplementary Fig. 4c

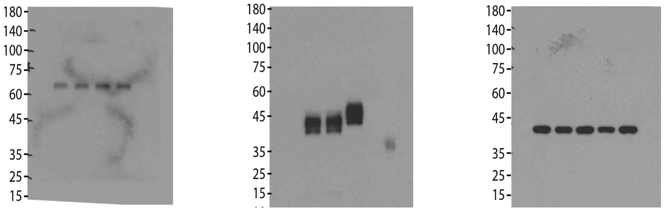

Supplementary Fig. 4e

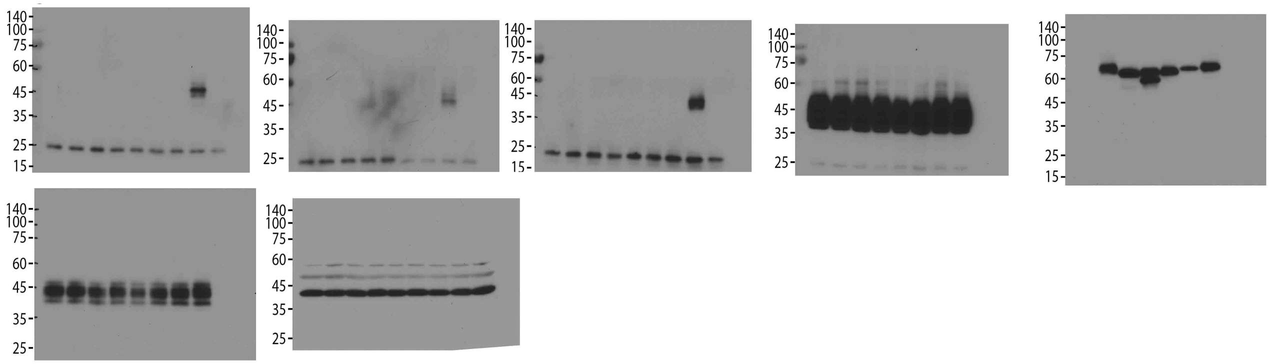

Supplementary Fig. 4f

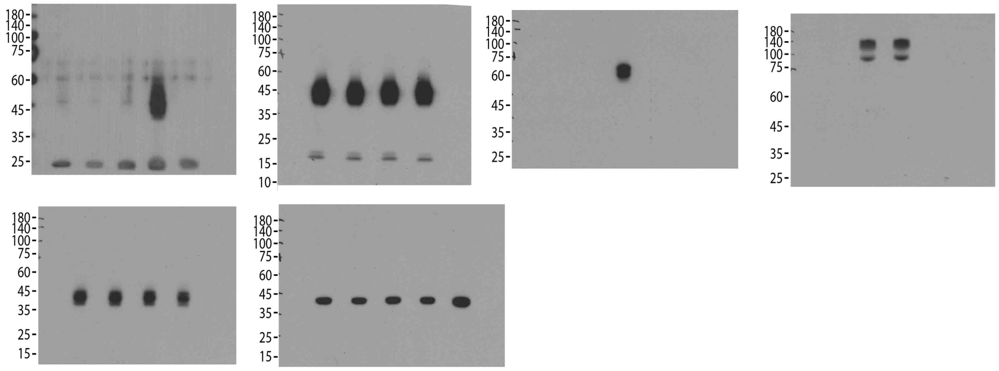

Supplementary Fig. 6c

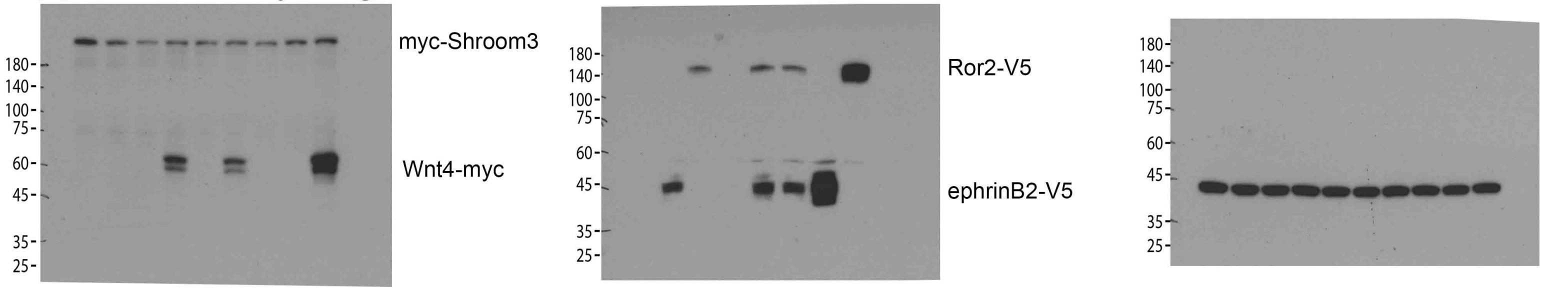

Supplementary Fig. 6d

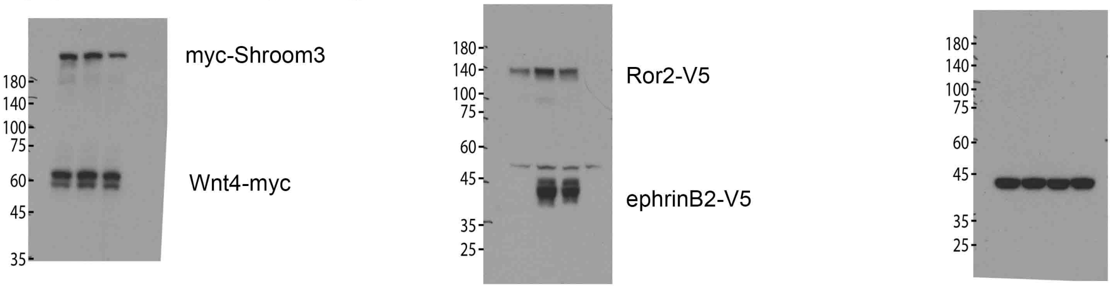

Supplementary Fig. 6e

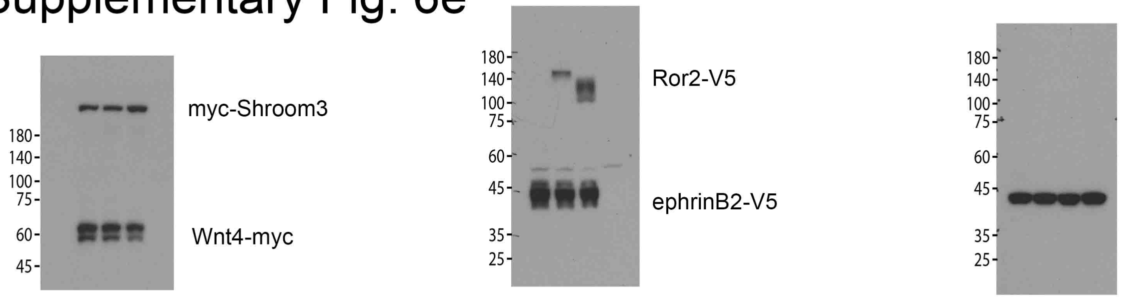

Supplementary Fig. 6f

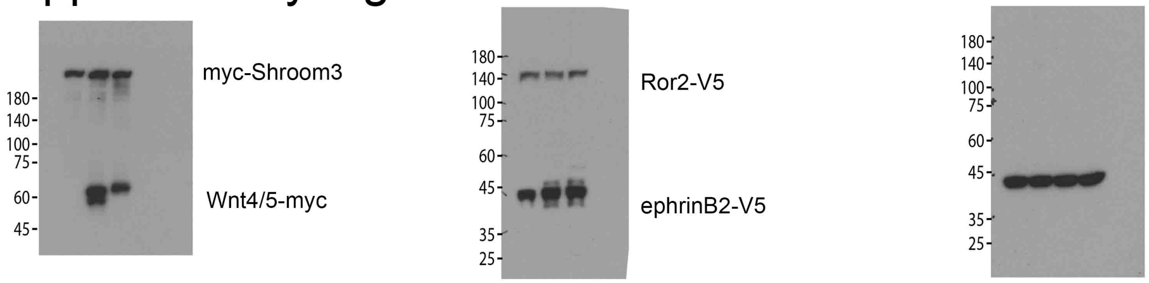

Supplement: Supplementary file 13 — Source Data [file 41467_2023_35991_MOESM13_ESM.zip › Source Data 2.pdf]
